# Multi-omics analysis reveals the attenuation of the interferon pathway as a driver of chemo-refractory ovarian cancer

**DOI:** 10.1016/j.xcrm.2025.102316

**Published:** 2025-08-29

**Authors:** Daria Afenteva, Rong Yu, Anna Rajavuori, Marina Salvadores, Inga-Maria Launonen, Kari Lavikka, Kaiyang Zhang, Anna Pirttikoski, Giovanni Marchi, Sanaz Jamalzadeh, Veli-Matti Isoviita, Yilin Li, Giulia Micoli, Erdogan Pekcan Erkan, Matias M. Falco, Daniela Ungureanu, Alexandra Lahtinen, Jaana Oikkonen, Sakari Hietanen, Anna Vähärautio, Inderpreet Sur, Anni Virtanen, Anniina Färkkilä, Johanna Hynninen, Taru A. Muranen, Jussi Taipale, Sampsa Hautaniemi

**Affiliations:** 1 Research Program in Systems Oncology, Research Programs Unit, Faculty of Medicine, University of Helsinki, Helsinki, Finland; 2 Department of Medical Biochemistry and Biophysics, Karolinska Institutet, Stockholm, Sweden; 3 Department of Obstetrics and Gynecology, University of Turku and Turku University Hospital, Turku, Finland; 4 Department of Obstetrics and Gynecology, Wellbeing Services County of Central Finland, Jyväskylä, Finland; 5 Genome Data Science, Institute for Research in Biomedicine (IRB Barcelona), Barcelona Institute of Science and Technology, Barcelona, Spain; 6 Disease Network Unit, Faculty of Biochemistry and Molecular Medicine, University of Oulu, Oulu, Finland; 7 Foundation for the Finnish Cancer Institute, Helsinki, Finland; 8 Department of Pathology, University of Helsinki and HUS Diagnostic Center, Helsinki, Finland; 9 Department of Obstetrics and Gynecology, Clinical Trials Unit, Comprehensive Cancer Center, Helsinki University Hospital, Helsinki, Finland; 10 Institute for Molecular Medicine Finland, Helsinki Institute for Life Sciences, University of Helsinki, Finland; 11 Applied Tumor Genomics Research Program, Research Programs Unit, Faculty of Medicine, University of Helsinki, Helsinki, Finland; 12 Department of Biochemistry, University of Cambridge, Cambridge, UK

**Keywords:** platinum-based chemotherapy, chemoresistance, ovarian cancer, chemo-refractory cancer, interferon type I signaling, single-cell RNA sequencing, cyclic immunofluorescence, real-world data, prospective clinical cohort

## Abstract

Ovarian high-grade serous carcinoma (HGSC) is one of the deadliest gynecological malignancies, with 10%–15% of patients exhibiting primary resistance to first-line chemotherapy. To characterize the molecular drivers of chemo-refractoriness, we perform multi-omics profiling of treatment-naive biopsies from patients with refractory HGSC enrolled in the DECIDER observational trial. We demonstrate that chemo-refractory HGSC is characterized by diminished interferon type I (IFN-I) and enhanced hypoxia pathway activity, and baseline IFN-I activity in chemo-naive cancer is an independent prognostic factor. Single-cell RNA sequencing and spatial protein profiling analyses corroborate the importance of elevated IFN-I activity in response to chemotherapy. Importantly, *in vitro* experiments demonstrate that high levels of IFN-I signaling increase cell chemosensitivity to platinum in a cell-autonomous manner. Together, these findings indicate that the IFN-I pathway activity in HGSC cancer cells predicts response to first-line chemotherapy in HGSC, proposing the stimulation of the IFN-I response as a therapeutic strategy. The study is registered at ClinicalTrials.gov (NCT04846933).

## Highlights

• Low IFN-I activity in cancer cells is a marker of poor response in HGSC
• Refractory HGSC shows a lack of cancer cell subpopulation with high IFN-I response
• Exogenous interferon-alpha sensitizes cancer cells to cisplatin

## Graphical abstract

**Figure:**
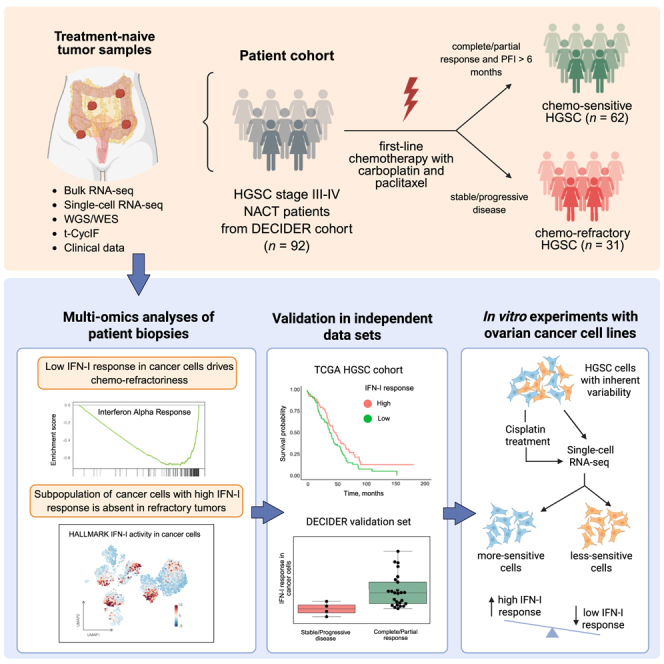

## Teaser

Afenteva et al. combine multi-omics, single-cell, and functional analyses to show that diminished type I interferon signaling in cancer cells underlies inherent platinum resistance in ovarian high-grade serous carcinoma. Exogenous interferon-alpha sensitizes cancer cells to cisplatin, nominating interferon pathway stimulation as a precision strategy for refractory disease.

## Introduction

Chemotherapy resistance is the leading cause of cancer-related deaths, representing the most critical unresolved challenge in oncology. The issue of chemotherapy resistance is particularly acute in ovarian high-grade serous carcinoma (HGSC), which is one of the deadliest gynecological malignancies. In 2020 alone, there were approximately 200,000 deaths from ovarian cancer worldwide.^1^ HGSC is typically diagnosed at an advanced stage when dissemination from ovaries and fallopian tubes, which are considered the site of origin, into the peritoneal cavity has already occurred,^2^^,^^3^^,^^4^ which hinders the effectiveness of treatments. Genomically, HGSC is a copy-number-driven cancer^5^ that is characterized by an almost 100% *TP53* mutation rate^6^ and high patient- and tissue-specific heterogeneity,^7^ making it particularly challenging to treat. For patients diagnosed at an advanced stage, the 5-year survival rate is <40%, largely due to the challenges posed by late-stage detection and metastasized disease.^8^^,^^9^

The standard of care for HGSC is cytoreductive surgery followed by platinum-taxane chemotherapy and possible maintenance therapy with anti-angiogenesis or poly (ADP-ribose) polymerase (PARP) inhibitor.^10^ The PARP inhibitors have significantly improved survival rates of patients with dysfunctional *BRCA1* or *BRCA2* and, more generally, patients with homologous recombination-deficient disease.^11^ Patients who have a low likelihood for optimal surgical cytoreduction at diagnosis (40%–50% of all patients with HGSC^12^) are referred to neoadjuvant therapy (NACT), which consists of 3–4 cycles of platinum-taxane chemotherapy followed by cytoreductive surgery, adjuvant chemotherapy, and possible maintenance therapy.^10^ Notably, 10%–15% of patients respond inconspicuously or not at all to NACT and cannot be operated. These patients with primarily chemo-refractory cancer have the worst prognosis among those with already poor-prognosis HGSC and are in dire need of effective treatment options.^13^

In our recent work, we revealed, using single-cell RNA sequencing (scRNA-seq) data from poorly responding patients with HGSC, that chemoresistance in HGSC is driven by pre-existing and induced cellular states, in particular, stress-associated cellular state.^14^ Herein, our goal is to further characterize the molecular drivers of chemo-refractoriness using a prospective subcohort of 31 patients with primarily chemo-refractory HGSC who belong to the DECIDER trial.^7^ We employed multi-omics data from these patients, including whole-genome sequencing (WGS), bulk RNA sequencing (RNA-seq) and scRNA-seq, and spatial data from highly multiplexed images, to discover and validate molecular drivers of primarily chemo-refractory disease. Our results show that the platinum response is associated with baseline interferon type I (IFN-I) pathway activity, opening the avenue for personalized modification of primary chemotherapy for patients with primarily chemo-refractory HGSC.

## Results

### Patient characteristics

**Figure 1:**
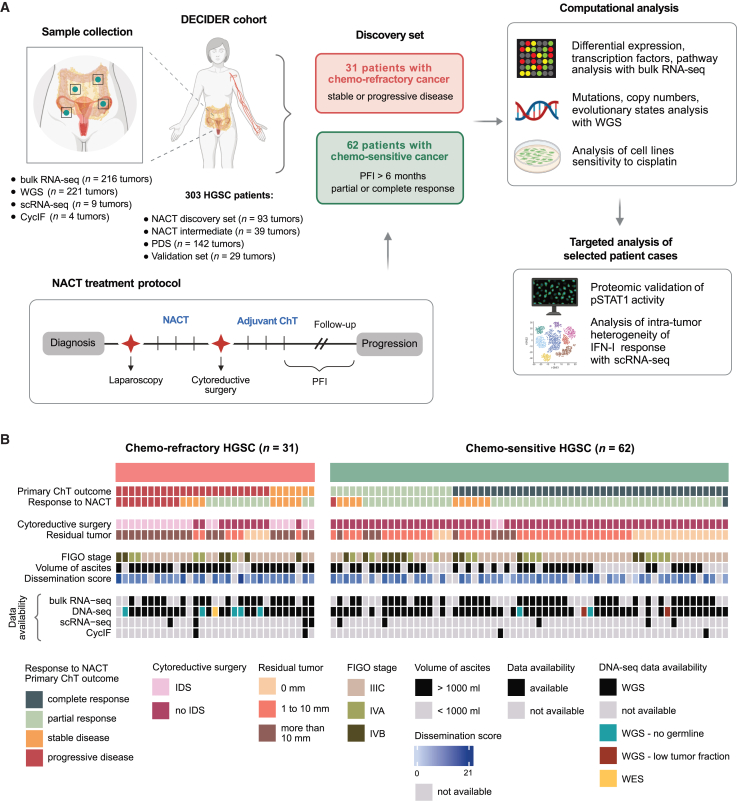
Study design and clinical characteristics of patients with chemo-refractory and chemo-sensitive HGSC from the DECIDER cohort (A) Schematic representation of sampling procedure and study design. DECIDER cohort comprised patients treated with primary debulking surgery (PDS) and neoadjuvant chemotherapy (NACT), including patients with chemo-refractory (*n* = 31) and chemo-sensitive (*n* = 62) HGSC in the discovery set. (B) Clinical characteristics of patients with chemo-refractory (left) and chemo-sensitive (right) HGSC included in the discovery set and data availability. See also Figure S1.

We established a subcohort of 31 NACT-treated patients with chemo-refractory HGSC from the prospective, longitudinal, multi-region, observational DECIDER trial (Multi-Layer Data to Improve Diagnosis, Predict Therapy Resistance and Suggest Targeted Therapies in HGSOC; ClinicalTrials.gov identifier NCT04846933) (Figures 1A, 1B, and S1 and STAR Methods). The chemo-refractory disease was defined as a stable or progressive disease after primary therapy according to the RECIST 1.1 criteria.^15^ Patients who did not receive at least two cycles of chemotherapy were excluded.

For comparison analyses, we selected 62 chemo-sensitive patients with similar baseline clinical characteristics, including the same treatment strategy but at least partial response to primary therapy and platinum-free interval, which is calculated from the last dose of platinum to first disease progression, exceeding 6 months (Figures 1A, 1B, and S1 and STAR Methods). The patient characteristics are shown in Table S1. Patients with refractory HGSC had a higher disease burden at the time of diagnosis based on a higher dissemination score^16^ (Fisher’s exact test *p* = 0.026) and more frequently detected large volumes of ascites (Fisher’s exact test *p* = 0.024) (Figure 1B; Table S1).

Diagnostic biopsies were available for 83 patients in the discovery set (Figure 1B). Bulk RNA-seq was performed on fresh frozen biopsies from 20 refractory and 38 chemo-sensitive tumors, a subset of which were selected for scRNA-seq (*n* = 9) (Figure S1). WGS data from tumor and germline reference samples were available from 23 patients with chemo-refractory cancer and 50 patients with chemo-sensitive cancer. Cyclic immunofluorescence (CycIF) was performed on four biopsies (Figure S1).

### Genomic landscape of chemo-refractory and chemo-sensitive tumors

**Figure 2:**
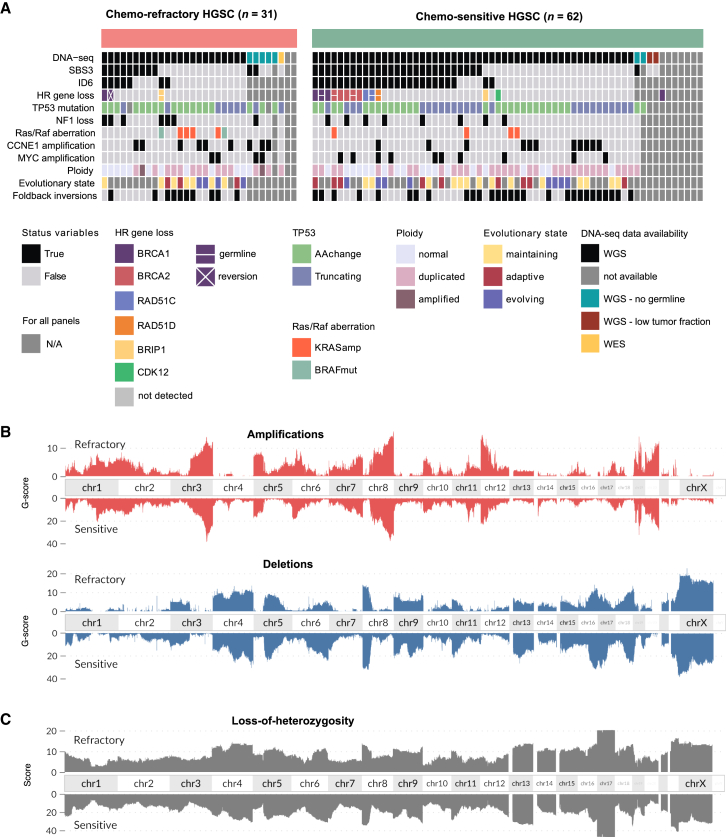
Genomic landscape of chemo-refractory and chemo-sensitive HGSC (A) Summary of genomic oncogenic events for the chemo-refractory (*n* = 31) and chemo-sensitive (*n* = 62) groups. (B) Amplifications (red) and deletions (blue) across all chromosomes in chemo-refractory patients, with genomic alterations plotted relative to a reference genome of patients with chemo-sensitive HGSC, revealing similar copy-number landscapes. (C) LOH across the genome of chemo-refractory and chemo-sensitive tumors. The G-score was calculated as the total magnitude of aberrations (logR) across the genome.^17^

We compared mutations, copy numbers, structural variants, and mutational signatures using WGS data from treatment-naive chemo-refractory and chemo-sensitive tumors. All patients had dysfunctional *TP53*, and there were no significant differences in mutations of homologous recombination repair genes, foldback inversions, and homologous recombination deficiency-associated somatic mutational signatures SBS3 and ID6 (Figure 2A; Table S1). Germline mutations of *BRCA1/2* (*n* = 3) and *RAD51C/D* (*n* = 2) were detected only in patients with chemo-sensitive HGSC, whereas a reversion mutation of *BRCA1* was detected in one patient with chemo-refractory HGSC.

Established oncogenic driver aberrations, such as amplifications of *KRAS*, *MYC*, and *CCNE1* and loss of *NF1*, were detected at comparable frequencies in chemo-refractory versus chemo-sensitive tumors (*KRAS* 14% vs. 8%, *MYC* 14% vs. 19%, *CCNE1* 32% vs. 21%, and *NF1* 25% vs. 15%; all false discovery rate [FDR]-adjusted Fisher’s exact *p* > 0.60) (Figure 2A). Oncogenic *BRAF* class III kinase-impaired mutations were identified in two patients with refractory cancer, while none were found in patients with sensitive cancer. Notably, within the entire DECIDER cohort (*n* = 221 patients with WGS data), there were two additional patients with *BRAF* class III mutations: one patient whose primary NACT was canceled after one cycle due to lack of response and another patient with poor survival who was treated with primary debulking surgery and thus not included in the discovery analysis. Whole-genome duplications were detected in more than half of the samples with no marked distinction between the two groups (Figure 2A). Additionally, the copy number profiles for gains, losses, and allelic imbalance were strikingly similar between the groups (Figure 2B). The loss of heterozygosity (LOH) of the entire length of chromosome 17 was observed in all cancer samples (Figure 2C). Tumor evolutionary states describing subclonal and site-associated heterogeneity were uniformly represented in the groups, as shown in the interactive GenomeSpy visualization of the genomic landscape of all samples^7^^,^^18^ (https://csbi.ltdk.helsinki.fi/p/chemorefractory/).

### Low-interferon, high-hypoxia cell state is associated with chemo-refractoriness and prognosis

To identify processes that drive chemo-refractory phenotype, we first decomposed bulk RNA-seq data to cancer, immune, and stromal components with PRISM^19^ and performed a differential expression analysis (DEA) for the cancer component of chemo-refractory and chemo-sensitive tumors (Figures S2A–S2D and STAR Methods). Leveraging gene-level statistics from DEA, we conducted pathway activity inference using PROGENy,^20^ evaluating enrichment scores for 14 pathways, and identified the perturbed transcription factors from transcriptomic data using CollecTRI^21^ (Figure S2A and STAR Methods).

**Figure 3:**
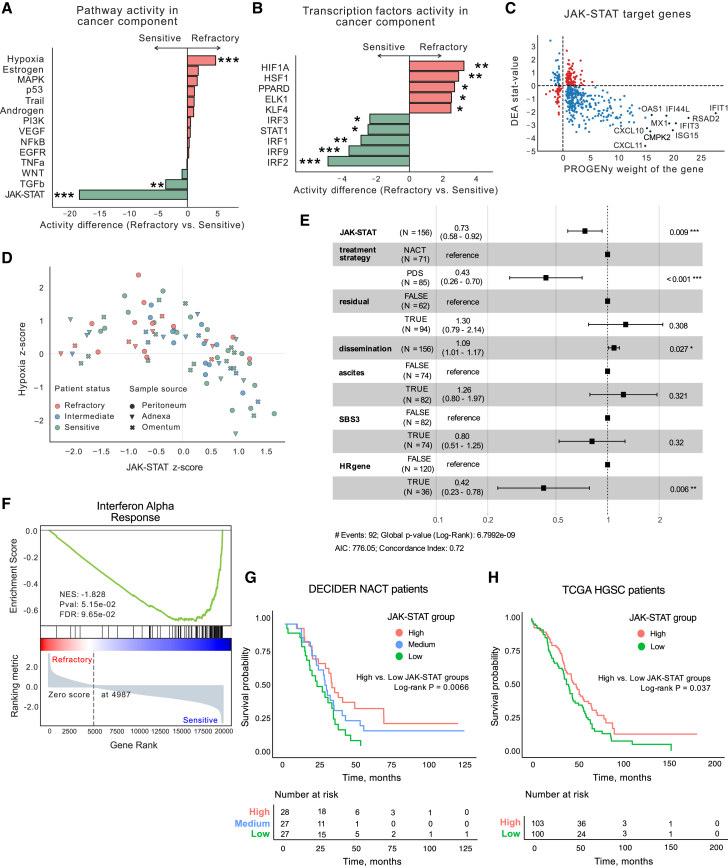
Pathways and transcription factors associated with chemo-refractoriness (A) Pathway activity differences between chemo-sensitive (*n* = 38) and chemo-refractory (*n* = 20) HGSC tumors, calculated with PROGENy in the cancer component from decomposed bulk RNA-seq data, show significant down-regulation of JAK-STAT and transforming growth factor β pathways, along with upregulation of the hypoxia pathway in refractory patients. *p* values were derived from two-sided t-statistics (multivariate linear model) in decoupleR and adjusted by Benjamini-Hochberg (BH) FDR. ∗*p* < 0.01, ∗∗*p* < 0.001, ∗∗∗*p* < 0.00001. (B) Differential activity of transcription factors between chemo-sensitive (*n* = 38) and chemo-refractory (*n* = 20) HGSC tumors calculated with CollecTRI on the cancer component from decomposed bulk RNA-seq data, highlighting lower activity of interferon regulatory factors *IRF2*, *IRF9*, and *IRF1* and higher *HIF1A* activity in refractory patients. The top 5 significantly up- and down-regulated transcription factors are presented. *p* values were derived from two-sided t-statistics (univariate linear model) in decoupleR and adjusted by BH FDR. ∗*p* < 0.01, ∗∗*p* < 0.001, ∗∗∗*p* < 0.00001. (C) Scatterplot depicting the weight of the JAK-STAT target genes (*x* axis) and their stat-value (*y* axis) derived from differential expression analysis (DEA) of the cancer component of chemo-refractory (*n* = 20) versus chemo-sensitive (*n* = 38) tumors. (D) Correlation plot of hypoxia and JAK-STAT *Z* scores calculated for cancer component across samples from patients with chemo-refractory (*n* = 20), intermediate (*n* = 24), and chemo-sensitive (*n* = 38) HGSC from the DECIDER cohort, indicating an inverse relationship. Data points are color-coded by patient status and symbol-coded by tissue origin. One representative treatment-naive sample from solid metastatic or intra-abdominal tissues with the lowest JAK-STAT score was taken per patient (Table S3 and STAR Methods). (E) Forest plot of multivariable Cox proportional hazards model showing the prognostic significance of the JAK-STAT score for overall survival (*n* = 156 patients) in NACT and PDS patients from the DECIDER cohort. One representative treatment-naive sample from solid metastatic or intra-abdominal tissues with the lowest JAK-STAT score was taken per patient (STAR Methods). The residual was classified as TRUE when the residual tumor after cytoreductive surgery was more than 0 mm. HRgene indicates the presence of a mutation in the homologous recombination deficiency (HRD)-related genes. SBS3 indicates the HRD status of a patient according to the SBS3 mutational signature. HRs and 95% CIs were estimated for each covariate, with *p* values derived from the Wald test. ∗*p* < 0.05, ∗∗*p* < 0.01, ∗∗∗*p* < 0.001. (F) GSEA indicating significant enrichment of the interferon alpha response pathway, with lower activity in the cancer component of chemo-refractory tumors, highlighted by the negative normalized enrichment score (NES). The BH FDR was used to adjust the *p* values. (G) Kaplan-Meier overall survival curves for the DECIDER NACT patients (*n* = 82), stratified into high, intermediate, and low JAK-STAT pathway activity groups based on PROGENy scores derived from bulk RNA-seq data. Patients in the JAK-STAT high-activity group (*n* = 28) displayed significantly longer overall survival than those in the low-activity group (*n* = 27; log rank *p* = 0.007). (H) Kaplan-Meier overall survival curves for the TCGA HGSC cohort (*n* = 303 patients). For clarity, only the JAK-STAT high- (*n* = 103) and low-activity (*n* = 100) groups are displayed. Patients in the high-activity group displayed significantly longer overall survival than those in the low-activity group (log rank *p* = 0.037). See also Figure S2.

The largest activity difference was observed in the JAK-STAT pathway (FDR-adjusted *p* < 1e−16), primarily due to the reduced expression of more than 77% of its positive targets, such as *CXCL11*, *CMPK2*, and *ISG15* (Figures 3A–3C). The most active pathway in chemo-refractory samples in comparison to chemo-sensitive samples was the hypoxia pathway (FDR-adjusted *p* < 1e−6) (Figure 3A), regulated by *HIF1A*-dependent transcription (FDR-adjusted *p* = 2.4e−4) (Figures 3B and S2E). Interestingly, we observed that low JAK-STAT activity and high hypoxia were inversely related phenotypes (Spearman correlation coefficient *ρ* = −0.59, *p* = 6.1e−9) (Figure 3D and Data S1).

To test whether the JAK-STAT pathway has an association with patient survival, we calculated PROGENy scores for the JAK-STAT pathway for all patients with bulk RNA-seq data from the DECIDER cohort (*n* = 156) and fitted the multivariable Cox proportional hazards model (Figure 3E and STAR Methods). The JAK-STAT score was prognostic for overall survival (hazard ratio [HR] = 0.73, 95% confidence interval [CI] = 0.58–0.92, *p* = 0.009). For NACT patients (*n* = 82) stratified into three groups by JAK-STAT activity scores, Kaplan-Meier survival analysis (Figure 3G) revealed a survival advantage in the high-activity group over the low-activity group (log rank *p* = 0.007). We also validated this association in The Cancer Genome Atlas (TCGA) cohort (*n* = 303), where the same stratification showed a similar survival benefit for the JAK-STAT high-activity group (Figure 3H; log rank *p* = 0.037). In the validation set of 29 additional DECIDER patients, JAK-STAT activity scores were significantly lower in the four patients with stable or progressive disease after primary chemotherapy than in the 25 patients with partial or complete response (Figure S2G; two-tailed Student’s t test *p* = 0.003).

To identify the perturbed axis of the JAK-STAT pathway in chemo-refractory tumors, we analyzed the activity of transcription factors, which revealed that interferon regulatory factors *IRF2*, *IRF9*, and *IRF1* had lower activity in refractory compared to the chemo-sensitive tumors (Figure 3B). The reduction in *IRF9* activity, a pivotal component of the IFN-stimulated gene factor-3 complex alongside *STAT1* and *STAT2*, directly implicates suppressed IFN-I response. The attenuation of this pathway in the chemo-refractory tumors was further evidenced by decreased expression levels of the *IRF9*-regulated genes *CXCL10*, *IFIT3*, *OAS1*, *IFIT1*, and *STAT1* (Figure S2F). Furthermore, the gene set enrichment analysis^22^ (GSEA) of the Molecular Signatures Database^23^ Hallmark collection indicated the interferon alpha response pathway as the most enriched (FDR-adjusted *p* = 0.096, normalized enrichment score = −1.828) in the genes ranked by the *t*-statistic (Figure 3F). To evaluate whether IFN-I pathway activity itself was associated with overall survival, we calculated Hallmark interferon alpha response enrichment scores and found that lower IFN-I activity was associated with shorter overall survival in the DECIDER cohort (HR = 0.94, 95% CI = 0.88–1.00, *p* = 0.046). Notably, the proportions of immunoreactive, mesenchymal, proliferative, and differentiated TCGA subtypes did not differ between chemo-refractory and chemo-sensitive tumors (chi-square test, *p* = 0.44). These analyses demonstrate that the IFN-I response is suppressed in patients with chemo-refractory HGSC at the time of diagnosis.

### Genomic perturbations do not explain reduced IFN-I activity in chemo-refractory patients

To investigate whether genetic aberrations are responsible for the attenuated IFN-I signaling in patients with chemo-refractory HGSC, we conducted an in-depth analysis of somatic aberrations of the genes implicated in the IFN-I response cascade (Figures S3A–S3C). The genomic landscape was devoid of loss-of-function mutations, except for a deletion in the IFN-alpha/epsilon and *CDKN2A/B* locus of one patient (Figure S3B). While our cohort is underpowered to rule out rare (<13%) genomic drivers, our results suggest that pervasive genomic alterations of the IFN-I axis are uncommon in chemo-refractory HGSC.

### scRNA-seq and spatial protein profiling of the IFN-I activity confirm its association with chemo-refractoriness

**Figure 4:**
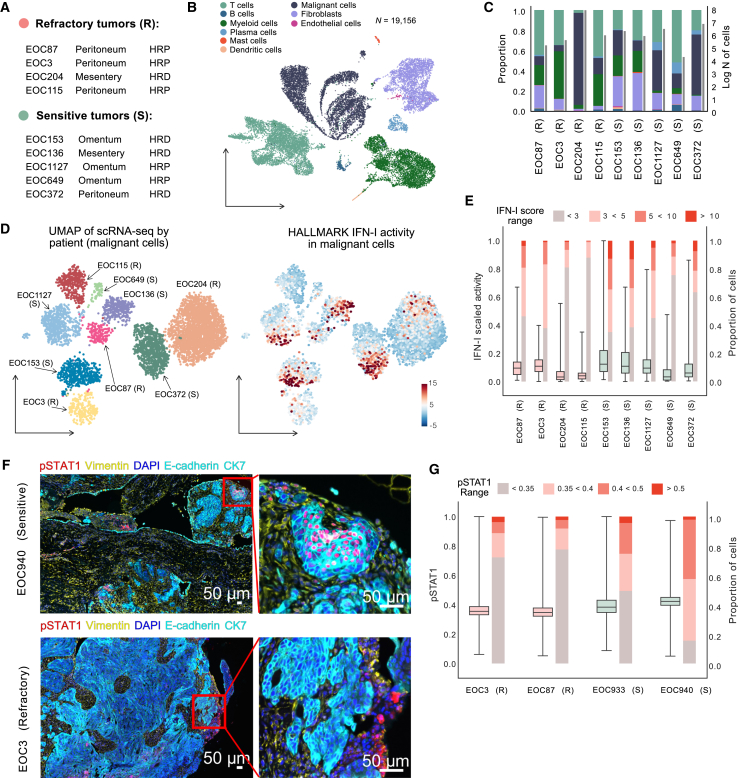
scRNA-seq and CycIF analyses of selected patients reveal the heterogeneity of IFN-I activity in chemo-sensitive patients (A) Overview of scRNA-seq data from solid metastatic tissues from four refractory (R) and five chemo-sensitive (S) HGSC tumors. Homologous recombination deficiency status was defined by the SBS3 mutational signature. (B) Uniform manifold approximation and projection (UMAP) of 19,156 scRNA-seq profiles from nine patients colored by cell type. (C) Proportions of different cell types (left axis) and logarithmic number of cells (right axis) per tumor. (D) UMAP of 5,449 malignant cells colored by the patient (left) and by the level of IFN-I activity calculated with DecoupleR (right). (E) Boxplots illustrating normalized IFN-I pathway activity scores in individual cells within each tumor, showing a broader spread of activity in chemo-sensitive tumors. Bar plots show the proportion of cells with IFN-I activity scores within a specific range for every tumor. Data are presented as median ± interquartile range (IQR) (box: Q1–Q3; whiskers: minimum and maximum). (F) Tissue cyclic multiplex immunofluorescence (t-CycIF) imaging of FFPE tumor samples from the omentum and peritoneum of chemo-refractory (EOC3) and chemo-sensitive (EOC940) tumors, respectively, with pSTAT1 expression indicating IFN-I activity. Scale bars, 50 μm. (G) Boxplots with pSTAT1 expression in malignant cells across chemo-refractory (EOC3 and EOC87) and chemo-sensitive (EOC933 and EOC940) patient samples. Bar plots show the proportion of cells with pSTAT1 expression within a specific range for every tumor. Data are presented as median ± IQR (box: Q1–Q3; whiskers: minimum and maximum). See also Figures S4 and S5.

We then characterized the IFN-I activity and heterogeneity using single-cell transcriptomic and spatial data from highly multiplexed images. scRNA-seq data were acquired from four refractory and five chemo-sensitive tumors (Figures 4A and S4C; Table S4). Employing a tiered clustering approach,^14^ we first cataloged 19,156 high-quality cells, including malignant (*n* = 5,449), immune (*n* = 10,475), and stromal cells (*n* = 3,232). Using canonical markers, we then classified stromal cells into endothelial cells and fibroblasts, and immune cells into T cells, B cells, plasma cells, mast cells, dendritic cells, and myeloid cells (Figures 4B, 4C, and S4A and STAR Methods). Immune and stromal cells clustered by cell identity across all patients, while malignant cells clustered by patient origin, highlighting the high inter-patient heterogeneity among tumor samples (Figures S5A and S5B). We detected no differences in the proportions of cell types between refractory and chemo-sensitive HGSC (Figure S4B).

Building on previous work that identified pre-existing cellular states driving chemoresistance in HGSC,^14^ we mapped each malignant cell to one of the predefined states (Figure S4G). The C3_4 cellular state, enriched for IFN signaling, was more abundant in chemo-sensitive samples (FDR-adjusted *p* = 0.017), while the C7 cluster, associated with stress signaling, showed higher proportions in refractory samples (FDR-adjusted *p* = 0.046) (Figure S4H).

To further evaluate the intra-patient variability of the IFN-I activity, we computed the IFN-I pathway score for every single cell and observed distinct patterns of its activation across various cell types (Figure S4E). The IFN-I pathway exhibited elevated activity in malignant cells of the patients with chemo-sensitive cancer compared to the patients with refractory cancer, corroborating the bulk RNA-seq results (generalized estimating equations [GEE] regression, *p* < 1e−9; patient-level permutation test, *p* = 0.038) (Figures 4D and S4D). Furthermore, we observed greater variability in IFN-I activity in the sensitive samples (Levene’s test, *p* < 1e−9), primarily driven by a subset of high-activity cells not seen in refractory samples (Figure 4E). We also evaluated transcription factor activities associated with IFN-I signaling. In line with our bulk RNA-seq findings, *IRF2* and *IRF9* transcription factors demonstrated significantly higher activity in the diagnostic samples of chemo-sensitive tumors (GEE regression, FDR-adjusted *p* = 0.006 and FDR-adjusted *p* = 0.021, respectively) (Figure S4F). Furthermore, consistent expression of eight IFN-stimulated genes across all nine tumors highlights the reproducibility of IFN-I markers across samples, while their levels are lower in the refractory tumors (Figure S4J).

We next performed a single-cell analysis of formalin-fixed paraffin-embedded (FFPE) tumor samples from omental and peritoneal regions of two refractory and two sensitive tumors using multiplexed CycIF (tissue CycIF [t-CycIF]) imaging.^24^ We annotated approximately 1.4 million cells into cancer, immune, and stromal cells using TRIBUS.^25^ We assessed the state of IFN-I pathway activity by quantifying the expression levels of its indicator phospho-STAT1 (pSTAT1). Spatially, the pSTAT1-positive cancer cells formed clusters in the chemo-sensitive tumors, whereas similar colocalization was not observed in the chemo-refractory tumors, where the great majority of cancer cells were negative for pSTAT1 (Figures 4F and 4G). In line with the scRNA-seq results, pSTAT1 expression was significantly higher in cancer (GEE regression, FDR-adjusted *p* < 0.001) and stromal compartments (GEE regression, FDR-adjusted *p* = 0.003) of chemo-sensitive tumors, with no significant difference in the immune compartment (GEE regression, FDR-adjusted *p* = 0.182) (Figure S4I).

### Platinum response of ovarian cancer cell lines is associated with IFN-I signaling

**Figure 5:**
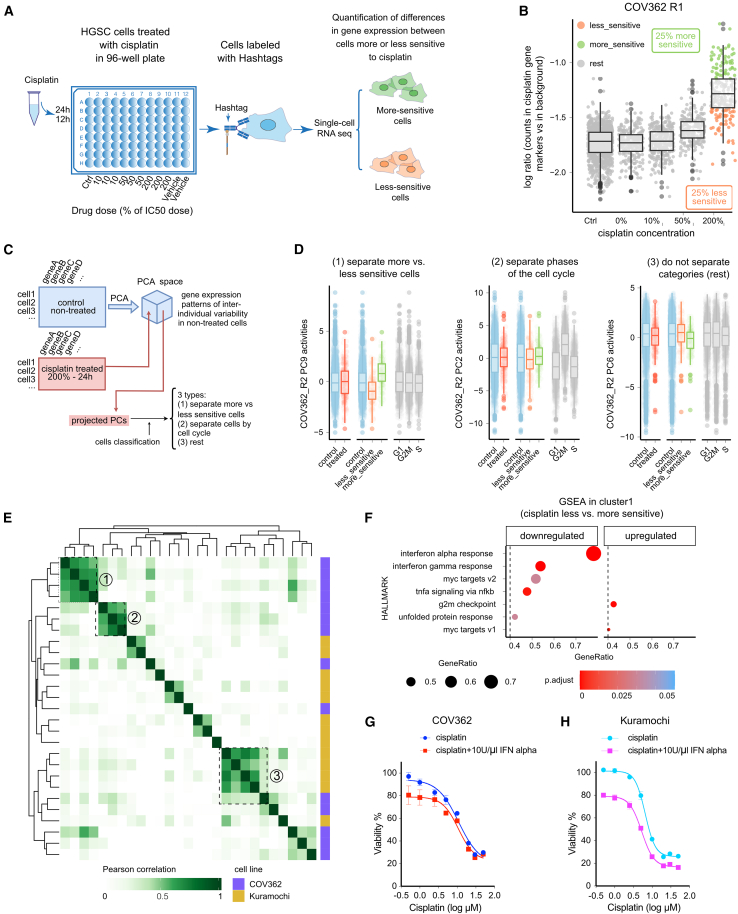
scRNA-seq in HGSC cell lines shows that IFN-I response determines platinum-based drug resistance (A) Workflow of the scRNA-seq combined with cell hashing in cell lines. (B) Based on the CisSenScore, the treated cells were separated into “more-sensitive” (top 25%, green) and “less-sensitive” cells (bottom 25%, orange) in COV362 cells. R1 refers to one of the experiment replicates. Data are presented as median ± IQR (box: Q1–Q3; whiskers: furthest points within 1.5× IQR). (C) Schematic of the data analysis pipeline. The non-treated cells underwent PCA to extract gene expression patterns of inter-individual variability, and the treated cells were projected into the PC space to be classified into three types based on the CisSenScore. (D) Three types of PCs in treated cells of the COV362_R2 experiment: (1) able to separate more-sensitive vs. less-sensitive cells; (2) able to separate different phases of the cell cycle; (3) not able to separate. Data are presented as median ± IQR (box: Q1–Q3; whiskers: furthest points within 1.5× IQR). (E) Heatmap depicting pairwise similarities between gene expression patterns of inter-individual variability identified in non-treated cells across replicates. Hierarchical clustering identified 3 clusters indicated by dashed boxes and numbers. (F) GSEA scores for the Hallmark gene sets (with *p* adjusted < 0.05 and GeneRatio >0.4) were calculated based on the gene expression signature in cluster 1 of the less-sensitive cells compared with the more-sensitive cells. Results from cluster 1 are shown. (G) Cell viability of COV362 cells treated with different concentrations of cisplatin-only or cisplatin combined with 10 U/μL IFN-alpha, *p* = 0.0009. (H) Cell viability of Kuramochi cells treated with different concentrations of cisplatin-only or cisplatin combined with 10 U/μL IFN-alpha, *p* < 0.0001. See also Figures S6–S8.

We then investigated the role of IFN-I signaling in platinum resistance in Kuramochi and COV362 cell lines (Table S2). The two cell lines were selected because they match clinical HGSC based on a high suitability score from a survey of 47 ovarian cancer cell lines.^26^ Furthermore, both cell lines show relatively high resistance to cisplatin, with COV362 being more resistant than Kuramochi.^27^ We performed scRNA-seq combined with cell hashing and determined transcriptomic profiles of the HGSC cells exposed to different concentrations of platinum (10%, 50%, and 200% of IC50) for different time intervals (24 h/12 h), with five replicates per cell line (Figure 5A). We first calculated the effect difference between platinum-treated and control cells by extracting the platinum gene expression signature from scRNA-seq data using Cohen’s *d* (Figure S6A and STAR Methods). The extracted platinum signatures showed a consistent correlation across the replicates (*n* = 5 for each cell line) (Figure S6A). In each replicate separately (*n* = 10 experiments), a platinum sensitivity score (CisSenScore, STAR Methods) for each cell was calculated, and based on it, the platinum-treated cells were classified into “more-sensitive” and “less-sensitive” groups (Figures 5B and S6B).

To identify cellular mechanisms influencing platinum response in an unbiased fashion, in each replicate separately (*n* = 10), we conducted principal component analysis (PCA) on scRNA-seq data from untreated cells, followed by projection of the expression data from treated cells onto the principal component (PC) state established by control cells’ expressions. This allowed the identification of PCs that capture the intrinsic variation in platinum response between individual cells in the culture population. Out of 100 PCs considered (the first 10 PCs with the highest variance in PCA of each experiment), we selected 27 PCs with a high variance effect size between more-sensitive and less-sensitive cells that do not separate cell cycle phases (Figures 5C and 5D and STAR Methods). Because these experiments are replicates (five per cell line), we can assess the robustness of the identified patterns by finding the agreement between PCs across replicates. For this, the resulting 27 PCs were clustered using hierarchical clustering, yielding three clusters (Figure 5E). Each cluster captures a robust (found in several replicates) transcriptional program that is linked to drug sensitivity. Analysis of the three clusters using GSEA^22^ showed that clusters 1 and 2 in the less-sensitive cells had a significantly down-regulated activity of the interferon alpha response pathway (Hallmark gene set), corroborating the IFN-I activity reduction in patients with chemo-refractory HGSC (Figures 5F and S6C–S6E). In COV362 cells, the top ten genes of cluster 1 showed significant variability in expression between individual cells of the culture. Furthermore, the cells with a high IFN-alpha response signature were more sensitive to platinum (Figure S8). In Kuramochi, the overall expression of these genes was low, and there was little variation between individual cells within the same culture (Figure S7), suggesting that the IFN pathway in Kuramochi cells is in an inactive state.

Next, we investigated the effect of IFN-alpha and platinum combination treatment on cell viability. Treatment with IFN-alpha alone had a negative effect on the growth of COV362 and Kuramochi cells (Figures S6F–S6G). For combination treatment, we selected a 10 U/μL concentration of IFN-alpha (Figure S6G). The combined treatment significantly reduced the cell viability compared with platinum-only treatment and showed additive effects of IFN-alpha and platinum in both COV362 (*p* = 0.0009) and Kuramochi (*p* < 0.0001) cell lines (Figures 5G and 5H). Taken together, these results indicate that the IFN-I signaling state of HGSC cells is intrinsically variable and that high levels of IFN-I signaling increase the chemosensitivity of the cells to platinum in a cell-autonomous manner.

## Discussion

Molecular processes driving chemo-refractory disease are poorly understood, and no clinically validated biomarkers or effective therapeutic strategies currently exist for patients with HGSC who do not respond to first-line chemotherapy. To address this urgent but unmet problem, we leveraged the DECIDER observational trial to obtain multi-omics data from patients with chemo-refractory and chemo-sensitive HGSC. By focusing on treatment-naive tumor biopsies, our bulk RNA-seq and complementary analyses were designed to capture the inherent drivers of primary chemoresistance rather than post-treatment adaptive changes driven by rare subclones.^28^

Our results show that the basal IFN-I pathway activity in cancer cells is a critical determinant of first-line chemotherapy response in HGSC. scRNA-seq and multiplexed imaging of patient tumor biopsies revealed a pronounced heterogeneity in IFN-I signaling across treatment-naive tumors. Specifically, higher baseline expression of IFN-stimulated genes correlated with improved platinum sensitivity, while low IFN-I activity was associated with poor outcome. Notably, the chemo-refractory tumors displayed a sparsity of IFN-responding cells, highlighting the importance of maintaining a substantial population of IFN-active cells to mediate effective response to treatment. Importantly, we showed that low IFN-I activity in cancer cells is an independent prognostic marker of poor response in patients receiving first-line platinum therapy. Furthermore, survival analysis demonstrated that lower IFN-I activity was also associated with shorter overall survival in the DECIDER cohort.

Recent work on molecular characterization of adaptation and acute response to PARP inhibitors suggested that IFN signaling upregulation is an early response.^29^ Over time, these highly adapted tumor states often evolve to silence IFN-induced pathways while concurrently activating *HIF1A*-mediated hypoxia signaling. Our findings—low IFN-I activity and high hypoxia-related signaling in primarily chemo-refractory HGSC—mirror this adaptive phenotype, explaining the limited efficacy of platinum-based regimens in these patients. While the association between pre-existing stress signature and poor survival has been reported previously,^14^ our findings highlight a significant link between pre-existing IFN signaling and chemosensitivity.

Within the confines of our cohort (*n* = 31 chemo-refractory tumors), we did not detect any recurrent genomic alteration that could account for primary resistance or explain diminished IFN-I activity. Given that this sample size affords 90% power only for drivers occurring in ∼13% or more of cases, rarer events cannot be excluded. Together with transcriptomic data, the findings point to a multifactorial basis that likely incorporates epigenetic and microenvironmental components in addition to patient-specific genomic changes. Unlike Chowdhury and colleagues,^30^ we neither observed the LOH of chromosome 17 nor an enrichment of wild-type *TP53* in patients with chemo-refractory HGSC; rather, every tumor in our cohort harbored a pathogenic *TP53* mutation accompanied by loss of the wild-type allele, except for a single case with borderline tumor fraction. We did replicate, however, their observation on the exclusivity of *BRAF* mutations in the refractory group. All four *BRAF*-mutated tumors across the full DECIDER cohort exhibited class III mutations and particularly poor survival, in line with data indicating that these kinase-impaired variants can retain partial RAS-dependent activity and confer resistance to platinum-based therapy.^31^ Although BRAF mutations occur in only ∼2.6% of HGSC,^31^ our data highlight the importance of comprehensive molecular profiling to identify the occasional patient who may respond to mitogen-activated protein kinase-targeted treatments.

The mechanistic relevance of IFN-I signaling was further supported by combination treatment experiments in ovarian cancer cell lines, which indicated that the IFN-mediated effect on platinum responsiveness is at least partly cancer cell intrinsic and does not solely depend on the tumor microenvironment. We studied two cell lines representative of the clinical scenarios observed in our patient cohort, COV362 and Kuramochi, which differed in their baseline IFN-I activity and, consequently, response to combination treatment. In COV362 cells, we detected baseline variability in IFN-I pathway activity within the cell population, with higher activity indicating a stronger response. Consistently, these cells displayed only a moderate increase in platinum sensitivity when the IFN-I pathway was activated by the addition of IFN-alpha. In contrast, Kuramochi cells exhibited uniformly low IFN-I pathway activity at baseline, and external stimulation with IFN-alpha significantly increased their sensitivity to cisplatin compared to COV362. This suggests that the majority of Kuramochi cells can benefit more from the combined effect (therefore causing a stronger effect in viability) than COV362, in which a subset of cells already have high expression of IFN-I response genes. These results suggest that enhancing IFN-I activity can effectively sensitize tumors with a sparse population of IFN-responding cells to platinum-based treatments. However, it is evident that IFN signaling is not the sole determinant of platinum sensitivity, as intrinsic differences in cisplatin responsiveness between cell lines like Kuramochi and COV362 reflect the involvement of additional resistance mechanisms.^32^^,^^33^^,^^34^^,^^35^^,^^36^ Our clustering approach also identified a specific resistance subset within Kuramochi cells (cluster 3, Figure S6D), further underscoring the complexity of resistance pathways in HGSC.

Our results suggest that patients with chemo-refractory HGSC may benefit from the stimulation of IFN-I activity to enhance their responsiveness to chemotherapy. Indeed, our combination experiment in ovarian cancer cell lines demonstrated that IFN-alpha exposure can potentiate cisplatin-induced cell death, consistent with a prior phase 2 trial in non-small cell lung cancer in which cisplatin plus IFN-alpha-2 produced notable response rates with acceptable toxicity.^37^ Various IFN-based strategies, such as macrophage^38^ reprogramming or IFN-epsilon^39^ treatment, are currently under development,^40^ and prospective clinical trials testing these approaches, in conjunction with platinum chemotherapy, are warranted. Historical experience with IFN-alpha in ovarian cancer provides important clues about exploiting the pathway clinically.^41^ Early phase 1/2 studies that delivered intraperitoneal IFN-alpha as salvage therapy, either alone or with intraperitoneal cisplatin, achieved transient control of malignant ascites and occasional pathological remissions, with a significantly higher response rate for the combination of intraperitoneal IFN-alpha and intraperitoneal chemotherapy.^42^^,^^43^ By contrast, a large randomized phase 3 trial that used low-dose subcutaneous IFN-alpha as maintenance after first-line chemotherapy showed no improvements in overall survival.^44^ These studies argue against IFN-alpha as a stand-alone maintenance therapy and support its concurrent use with platinum. In our findings, baseline IFN-I signaling emerges as a putative biomarker in treatment-naive HGSC, and our results support concurrent IFN-I stimulation during first-line platinum-taxane therapy rather than sequential or maintenance use.

In conclusion, we established IFN-I pathway activity in cancer cells as a key determinant of the response to the primary platinum-based chemotherapy, offering both a putative biomarker and a therapeutic target to overcome chemo-refractoriness in HGSC. By providing a detailed examination of the underrepresented patient population in ovarian cancer research, our results advocate for the urgent initiation of clinical trials to evaluate IFN-I modulation strategies for chemo-refractory patients.

### Limitations of the study

We acknowledge that the precise mechanism of IFN-I pathway suppression remains undetermined. Although the absence of recurrent genomic drivers points toward epigenetic or microenvironmental regulation, mechanistic dissection in immunocompetent pre-clinical models is still required. Our discovery cohort contained only 31 chemo-refractory tumors; under a Poisson model, this affords ∼90% power to detect recurrent alterations that occur in >13% of cases, and—with 47× median coverage—mutations present at < 15% variant-allele fraction in samples of <100% purity may have been missed. Additionally, although no subtype-specific effect was detected in the DECIDER cohort, the sample size may have limited power to reveal subtle IFN-I differences within individual HGSC molecular subtypes. Therefore, validation in larger, independent HGSC cohorts is essential to confirm the prognostic and therapeutic relevance of IFN-I signaling. Since the study focused on resistance to first-line chemotherapy, it remains an open question whether IFN-I also plays a key role in the acquired resistance observed in relapses.

## Resource availability

### Lead contact

Further information and requests for resources and reagents should be directed to and will be fulfilled by the lead contact, Sampsa Hautaniemi (sampsa.hautaniemi@helsinki.fi).

### Materials availability

This study did not generate new reagents or materials.

### Data and code availability

• Raw DNA sequencing data, raw bulk RNA-seq data, and raw scRNA-seq data have been deposited at the European Genome-phenome Archive and are publicly available as of the date of publication. Quantified signals for t-CycIF data have been deposited in Synapse and are publicly available as of the date of publication. Processed scRNA-seq data have been deposited in Gene Expression Omnibus and are publicly available as of the date of publication. Accession numbers are listed in the key resources table.
• All original code has been deposited at Zenodo at https://doi.org/10.5281/zenodo.15525180 and is publicly available as of the date of publication.
• Any additional information required to reanalyze the data reported in this paper is available from the lead contact upon request.

## Acknowledgments

We thank the patients and their families. This project received funding from the 10.13039/501100007601European Union’s Horizon 2020 Research and Innovation Program under grant agreement 965193 (DECIDER), the 10.13039/501100002341Research Council of Finland, the 10.13039/501100006306Sigrid Jusélius Foundation, the 10.13039/501100010711Cancer Foundation Finland, Cancerfonden (Sweden), and the 10.13039/501100004359Swedish Research Council. This study was co-funded by the 10.13039/501100000780European Union (ERC, SPACE 101076096). Views and opinions expressed are, however, those of the authors only and do not necessarily reflect those of the European Union or the 10.13039/501100000781European Research Council. Neither the 10.13039/501100000780European Union nor the granting authority can be held responsible for them. This program has been supported with an educational grant via the Gilead Nordic Fellowship Program. D.A. gratefully acknowledges support from 10.13039/501100007083Orion Research Foundation and Ida Montinin Säätiö. The authors wish to acknowledge the CSC-IT Center for Science (Finland) for computational resources. The Auria biobank (https://www.auria.fi/biopankki/) is acknowledged for delivering biobank samples and H&E images to our study. The authors also thank the core facility at NEO, BEA, Bioinformatics and Expression Analysis, which is supported by the board of research at the 10.13039/501100004047Karolinska Institutet and the research committee at the Karolinska Hospital. The results published in this article are in part based upon data generated by The Cancer Genome Atlas, managed by the NCI and National Human Genome Research Institute. Information about The Cancer Genome Atlas can be found at https://cancergenome.nih.gov. BioRender.com is acknowledged for providing icons and illustration software used as a basis for Figures 1A, S1, S2A, and S3A and a graphical abstract (https://biorender.com/xnic15b). We thank Dr. Kaisa Huhtinen for assisting in sample sequencing, Dr. Ann-Christin Ostwaldt for coordinating the DECIDER project, and Jenni Lahtinen for the management of laboratory operations. Open access publication was supported by the Helsinki University Library.

## Author contributions

D.A., R.Y., I.S., J.H., T.A.M., J.T., and S. Hautaniemi conceptualized the project. S. Hietanen and J.H. oversaw patient enrollment and coordinated the sample collection. A.R., V.-M.I., A. Virtanen, and J.H. reviewed clinical data. D.A., K.Z., and S.J. performed bulk RNA-seq sample processing and analysis. D.A., K.Z., A.P., E.P.E., M.M.F., and A. Vähärautio performed scRNA-seq sample processing and analysis. K.L., G. Marchi, Y.L., G. Micoli, A.L., J.O., and T.A.M. performed WGS sample processing and analysis. I.-M.L. and A.F. performed CycIF data processing and analysis. D.A. and T.A.M. performed and interpreted the omics computational analyses. R.Y., D.U., I.S., and J.T. designed and performed the cell line experiments. M.S. analyzed and interpreted results from the cell line experiments. D.A., R.Y., M.S., I.S., and T.A.M. wrote the draft of the manuscript. D.A., R.Y., A.R., M.S., I.-M.L., K.L., G. Marchi, G. Micoli, I.S., J.H., T.A.M., J.T., and S. Hautaniemi contributed to the writing of the manuscript. All authors reviewed and approved the manuscript.

## Declaration of interests

The authors declare no competing interests.

## Declaration of generative AI and AI-assisted technologies

During the preparation of this work, the authors used ChatGPT to improve clarity and language in the writing of the manuscript. After using this tool or service, the authors reviewed and edited the content as needed and take full responsibility for the content of the publication.

## STAR★Methods

### Key resources table

**Table:** 

REAGENT or RESOURCE	SOURCE	IDENTIFIER
**Antibodies**		

Anti-human CD298	BioLegend	Cat# 341712, RRID: AB_2876646
Anti-human β2-microglobulin	BioLegend	Cat# 316302, RRID: AB_492835
E-Cadherin (24E10) Rabbit mAb (Alexa Fluor ® 488 Conjugate)	Cell Signaling Technologies	Cat# 3199S; RRID: AB_10691457
Alexa Fluor ® 555 Anti-Cytokeratin 7 antibody [EPR17078]	Abcam	Cat# ab209601; RRID: AB_2728790
Vimentin (D21H3) XP ® Rabbit mAb (Alexa Fluor ® 555 Conjugate)	Cell Signaling Technologies	Cat# 9855S; RRID: AB_10859896
Vimentin (D21H3) XP ® Rabbit mAb (Alexa Fluor ® 750 Conjugate)	Cell Signaling Technologies	Cat# 69227S; RRID: AB_3331655
Phospho-Stat1 (Tyr701) (58D6) Rabbit mAb (Alexa Fluor ® 555 Conjugate)	Cell Signaling Technologies	Cat# 8183S; RRID: AB_10860600

**Biological samples**		

Fresh-frozen tissue samples	This paper	N/A
FFPE tissue section	This paper	N/A

**Chemicals, peptides, and recombinant proteins**		

Recombinant Human Interferon alpha 2a	R&D systems	Cat# 11100-1
Methyltetrazine-PEG4-NHS Ester	Click Chemistry tools	Cat# 1069-1
TCO-PEG4-NHS Ester	Click Chemistry tools	Cat# A137-2

**Critical commercial assays**		

CellTiter-Glo 2.0 Cell Viability Assay	Promega	Cat# G9242
Chromium Next GEM Single Cell 3′ Reagent Kits v3.1 (Dual Index)	10x Genomics	CG000317 Rev C
Chromium Single-Cell 3′ Reagent Kit v2.0	10x Genomics	CG00052 Rev F
Chemagic DNA Blood Kit Special	Perkin Elmer Inc.	CMG-1091
DNBSEQ (BGISEQ-500/MGISEQ-2000)	MGI Tech Co. Ltd.	https://en.mgi-tech.com/products/
HiSeq X Ten	Illumina	https://emea.support.illumina.com/sequencing/sequencing_instruments/hiseq-x.html
NovaSeq 6000	Illumina	https://emea.illumina.com/systems/sequencing-platforms/novaseq.html

**Deposited data**		

Raw DNA-seq data	EGA	EGAS00001006775
Raw bulk RNA-seq data	EGA	EGAS00001004714
Raw single-cell RNA-seq data	EGA	EGAS00001005010
Processed single-cell RNA-seq data	GEO	GSE300897
Processed t-CycIF data	Synapse	https://doi.org/10.7303/syn53283672
TCGA bulk RNA-seq data	Genomic Data Commons	https://portal.gdc.cancer.gov/

**Experimental models: Cell lines**		

COV362	Sigma-Aldrich	Cat# 07071910-1VL
Kuramochi	JCRB Cell Bank (NIBION)	Cat# JCRB0098

**Oligonucleotides**		

See Table S5 for the full list		N/A

**Software and algorithms**		

Anduril v2.0-7e2341d	Cervera et al.^45^	https://anduril.org
Affinity Designer	Serif	https://affinity.serif.com/en-us/
BioRender	BioRender	https://biorender.com/
Trimmomatics v0.32 and v0.33	Bolger et al.^46^	http://www.usadellab.org/
FastQC v0.11.4	Andrews^47^	https://www.bioinformatics.babraham.ac.uk/projects/fastqc/
BWA-MEM v0.7.12–41039	Li^48^	http://bio-bwa.sourceforge.net/
SAMtools		http://www.htslib.org/
Picard v2.6	Broad Institute	https://github.com/broadinstitute/picard
GATK v4.1.9.0	McKenna et al.^49^	https://gatk.broadinstitute.org/hc/en-us
ASCAT v2.5.2	van Loo et al.^50^	https://github.com/VanLoo-lab/ascat/releases
BEDtools v2.28.0	Quinlan,^51^ Quinlan & Hall^52^	https://bedtools.readthedocs.io/en/latest/
GISTIC v2.0.23	Mermel et al.^53^	https://bioinformaticshome.com/tools/cnv/descriptions/GISTIC.html
PRISM v0.9	Häkkinen et al.^19^	https://bitbucket.org/anthakki/prism/
CADD v1.6	Rentzsch et al.^54^	https://cadd.gs.washington.edu/download
ANNOVAR 20191024	K. Wang et al.^55^	https://annovar.openbioinformatics.org/
SePIA	Icay et al.^56^	https://anduril.org/sepia/
STAR v2.5.2b	Dobin et al.^57^	https://github.com/alexdobin/STAR
eXpressv1.5.1-linux_x86_64	Roberts & Pachter^58^	https://pachterlab.github.io/eXpress/
POIBM method	Holmström et al.^59^	https://doi.org/10.5281/zenodo.6122436
Kassandra deconvolution algorithm	Zaitsev et al.^60^	https://github.com/BostonGene/Kassandra
GRIDSS v2.13.2	Cameron et al.^61^	https://github.com/PapenfussLab/gridss
PURPLE v3.7.2	Cameron et al.^62^	https://github.com/hartwigmedical/hmftools/tree/master
PolyPhen2	Adzhubei et al.^63^	N/A
SIFT	Ng et al.^64^	N/A
BaSiC tool and ASHLAR algorithm	Muhlich et al.^65^	https://github.com/labsyspharm/ashlar
UNET	Ronneberger et al.^66^	https://lmb.informatik.uni-freiburg.de/people/ronneber/u-net/
TRIBUS	Kang et al.^25^	https://github.com/farkkilab/tribus
Cell Ranger v6.0.1 and v6.0.2	10x Genomics	https://www.10xgenomics.com/support/software/cell-ranger/latest
GenomeSpy	Lavikka et al.^18^	https://genomespy.app/
R v3.6.3 and higher	R Core Team	https://www.r-project.org
R package ggplot2 v3.5.2	CRAN	https://ggplot2.tidyverse.org/
R package pheatmap v1.0.12	CRAN	https://www.rdocumentation.org/packages/pheatmap/versions/1.0.12/topics/pheatmap
R package tidyverse v2.0.0	CRAN	https://www.tidyverse.org/
R package devtools v2.4.5	CRAN	https://devtools.r-lib.org/
R package ComplexHeatmap v2.23.1	Zuguang Gu et al.^67^	https://github.com/jokergoo/ComplexHeatmap
R package survival v3.8.3	CRAN	https://www.rdocumentation.org/packages/survival/versions/3.8-3
R package survminer v0.5.0	CRAN	https://github.com/kassambara/survminer
R package Seurat v4.0.1 and higher	Butler et al.^68^	https://satijalab.org/seurat/
R package DoubletFinder v2.0.4	McGinnis et al.^69^	https://github.com/chris-mcginnis-ucsf/DoubletFinder
Python v3.8.10	Python Software Foundation	https://www.python.org/
Python package pandas v1.5.3		https://pandas.pydata.org/
Python package numpy v1.23.5		https://numpy.org/
Python package scipy v1.9.8		https://scipy.org/
Python package seaborn v0.13.2		https://github.com/mwaskom/seaborn?tab=readme-ov-file
Python package matplotlib v3.7.4		https://github.com/matplotlib/matplotlib?tab=readme-ov-file
Python package scanpy v1.9.8	Wolf et al.^70^	https://github.com/scverse/scanpy
Python package decoupler v1.6.0	Badia-i-Mompel et al.^71^	https://github.com/saezlab/decoupler-py
Python package pydeseq2 v0.4.4	Muzellec et al.^72^	https://github.com/owkin/PyDESeq2
Python package gseapy v1.1.4	Zhuoqing et al.^73^	https://github.com/zqfang/GSEApy
Python package anndata v0.9.2	Wolf et al.^70^	https://github.com/dynverse/anndata
Python package statsmodels v0.14.1		https://github.com/statsmodels/statsmodels
Python package conorm v1.2.0		https://gitlab.com/georgy.m/conorm
Python package scimap	Nirmal et al.^74^	https://github.com/labsyspharm/scimap
GraphPad Prism 10.0.0	GraphPad	https://www.graphpad.com/scientific-software/prism/
Custom scripts related to this paper	This paper	https://doi.org/10.5281/zenodo.15525180

### Experimental model and study participant details

#### Study participants

The DECIDER study (Multi-Layer Data to Improve Diagnosis, Predict Therapy Resistance and Suggest Targeted Therapies in HGSOC; ClinicalTrials.gov identifier NCT04846933) fosters ongoing prospective recruitment of female patients diagnosed with HGSC at the Turku University Hospital, which started in 2010. All patients participating in the study gave their informed consent, and the study was approved by the Ethics Committee of the Hospital District of Southwest Finland (ETMK 145/1801/2015).

Clinical data on patient characteristics were collected from hospital records. For these analyses, we included patients diagnosed before 2022-03-31. The primary treatment strategy of all patients adhered to the ESMO guidelines,^10^ including either primary debulking surgery (PDS) followed by platinum-based adjuvant chemotherapy, or neoadjuvant chemotherapy (NACT), interval debulking surgery (IDS), and adjuvant chemotherapy. The chemo-refractory study group included patients from the NACT-treated arm and was defined by their outcome from primary therapy with either stable or progressive disease, according to the RECIST version 1.1 criteria.^15^ The platinum-free interval (PFI) of these patients was at highest 45 days, adhering to the ESMO definition of chemo-refractory^13^ HGSC with a slight concession for delayed progression detection. We excluded eleven patients from the analyses, most of whom did not receive adequate NACT treatment, comprising at least two cycles of chemotherapy, due to pre-existing medical conditions or severe side effects. The reference group consisted of patients with chemo-sensitive HGSC with similar baseline characteristics and treatment strategy but with either partial or complete response to primary therapy and a PFI of more than six months. This patient selection yielded 31 chemo-refractory and 62 chemo-sensitive cases. The full DECIDER cohort, including 39 additional NACT-treated patients with intermediate follow-up, 142 patients treated with PDS and adjuvant chemotherapy, and 29 patients from the validation set, was used to validate the findings from the discovery set (Figure S1; Table S1).

#### Cell lines and reagents

Ovarian cancer cell line COV362 (Sigma-Aldrich) was cultured in Dulbecco’s Modified Eagle’s Medium (Gibco) supplemented with 10% (vol/vol) fetal bovine serum (FBS, Thermo Fisher Scientific) and 1% Penicillin-Streptomycin antibiotics (Thermo Fisher Scientific). Ovarian cancer cell line Kuramochi (JCRB Cell Bank) was cultured in RPMI 1640 (Gibco) supplemented with 10% FBS and 1% Penicillin-Streptomycin antibiotics. All cells were cultured at 37°C under 5% CO_2_ and were Mycoplasma-free. Recombinant Human Interferon alpha 2a (IFN-alpha) was purchased from R&D systems. Cisplatin was purchased from Sigma-Aldrich.

### Method details

#### Sample preparation and selection

Tissue specimens from tumors were collected during diagnostic laparoscopy before treatment or palliative ascites removal and were subsequently subjected to pathological examination. Peripheral blood samples or buffy coat extracts were extracted in Auria Biobank for all patients for DNA extraction and genomic sequencing to serve as germline reference for identifying somatic genomic aberrations using Chemagic DNA Blood Kit Special (PerkinElmer Inc., USA) and Chemagic 360 instrument (PerkinElmer Inc., USA). For other samples, to extract DNA and RNA simultaneously, we utilized the Qiagen AllPrep kit (#80204). Slides of formalin-fixed paraffin-embedded (FFPE) tissue were stained with hematoxylin and eosin, scanned. A gynecopathologist (AVi) verified that all samples were indeed serous carcinomas. Tumor samples subjected to bulk and single-cell RNA-seq analyses originated from primary tubo-ovarian sites (ovaries and fallopian tubes) and solid metastatic sites (omentum, mesentery, and peritoneum). As many subsequent analyses required one sample per patient, we prioritized the samples from the solid metastatic sites. When multiple samples from solid metastatic sites were available for a patient, we selected the one with the highest abundance of EOC, according to PRISM.^19^ To compare bulk RNA-seq data from chemo-refractory and chemo-sensitive tumors, we utilized samples from fresh frozen solid tumor biopsies listed in Table S3.

#### Whole-genome and RNA sequencing

Tissue samples were extracted from fresh frozen tissue, and those with sufficient DNA/RNA content were sent to BGI (BGI Europe A/S, Denmark) or Novogene (Novogene Europe, UK) for library preparation and nucleotide sequencing. Whole-genome sequencing (WGS) was performed with either DNBSEQ (BGISEQ-500 or MGISEQ-2000, MGI Tech Co., Ltd., China), HiSeq X Ten (Illumina, USA), or NovaSeq 6000 (Illumina, USA) as 100bp or 150bp paired-end sequencing with a median coverage of 47x. RNA sequencing was performed using DNBSEQ, HiSeq X Ten, HiSeq 4000, or NovaSeq 6000 as 100bp or 150bp paired-end sequencing.

#### Bulk RNA-seq analysis

##### RNA-seq preprocessing

Bulk RNA sequencing (RNA-seq) reads were processed using the SePIA pipeline^56^ within the Anduril2^45^ framework, which provides a modular and reproducible platform for large-scale transcriptomic data analysis by integrating various bioinformatics tools, including Trimmomatic,^46^ STAR aligner,^57^ and eXpress.^58^ Trimmomatic^46^ v0.33 was used to trim low-quality bases as follows: (i) the first 12 bases were cropped due to uneven per-base sequence content; (ii) any leading bases with a quality score lower than 20 and any trailing bases with a quality score lower than 30 were removed; (iii) the reads were scanned with a 5-base wide sliding window, cutting when the average quality per base drops below 20; (iv) resulting sequences shorter than 20 bp were discarded. Trimmed reads were aligned to GRCh38.d1.vd1 with GENCODE v25 annotations via the STAR aligner^57^ v2.5.2b, allowing up to 10 mismatches. Transcripts per million (TPM) and gene-level effective counts were quantified using eXpress^58^ v1.5.1-linux_x86_64. We applied the POIBM method for batch-effect correction to gene-level read counts.^59^

##### Bulk RNA-seq decomposition

We employed the latent statistical framework PRISM to extract the sample composition, scale factors, and cell-type-specific whole-transcriptome profiles adapted to each transcriptomic sample.^19^ For the single-cell reference, we utilized single-cell RNA-seq data from HGSC, annotated for EOC, fibroblasts, immune cells, and other, encompassing data from eight patients and various anatomical sites. Raw read counts were utilized as input for the model. This approach enabled us to accurately generate EOC-specific gene-level read counts for each bulk RNA sample.

##### Differential expression analysis

Prior to differential expression analysis (DEA), we refined the matrix of raw read counts by filtering out genes that were not adequately profiled. The criteria for retaining genes in the analysis were as follows: (1) the gene must have a minimum of 15 total reads across samples within each comparison group, and (2) the gene must have at least 10 counts in at least some samples within each group. This filtering process resulted in an input matrix comprising 15,730 genes across 58 samples. For the DEA, we utilized the Python implementation of the DESeq2 framework pydeseq2^72^ v0.4.4. This analysis aimed to identify genes differentially expressed between the chemo-refractory and chemo-sensitive tumors, with SBS3 and refractory/sensitive status as design factors in our model. We incorporated a unique approach for determining size factors, as follows: we utilized scale factors derived for each RNA sample through the PRISM framework, multiplied by the EOC abundance, and normalized by the geometric mean across all samples.

##### TF and pathway activity inference

To assess the pathway activity, we employed the Python implementation of the DecoupleR^71^ framework v1.6.0 and retrieved the PROGENy^20^ model weights with *decoupler.get_progeny(top = 500)*. The multivariate linear model method *decoupler.run_mlm()* was used to infer the pathways enrichment scores. In addition to pathway activity scores, we also evaluated the activity scores of transcription factors (TF). For this purpose, we retrieved the CollecTRI^21^ gene regulatory network with *decoupler.get_collectri()*. For the inference of enrichment scores of TF, we utilized the univariate linear model method executed via *decoupler.run_ulm()*. We used the gene-level statistic incorporated in the stat-value of the DESeq2 output as input for this analysis.

##### GSEA

To infer functional enrichment scores, Gene Set Enrichment Analysis^22^ (GSEA) was conducted on the Trimmed Mean of M-values (TMM) normalized EOC expression profiles obtained using the Python package conorm v1.2.0. We utilized the Hallmark gene sets from the Molecular Signatures Database^23^ (MSigDB) collection for this analysis. The GSEA^22^ was performed using the gseapy Python library v1.1.4, applying the following parameters: ranking method set to 't_test,' a total of 1000 permutations for robust statistical assessment, and the inclusion criteria for gene sets were defined with a minimum of 15 genes and a maximum of 500 genes per set. We considered a pathway significantly enriched if Benjamini–Hochberg (BH)-adjusted *p* < 0.1.

##### Cell type abundance estimation

The Kassandra^60^ algorithm (tumor model) was used to evaluate the proportions of different cell types in the TME for each bulk RNA sample. TPM values obtained during the expression quantification phase served as the input data. When gene annotations were absent, zeroes were input to meet the algorithm’s input criteria.

#### Single-cell RNA analysis

##### Sample collection and data pre-processing

Tumor samples were collected at the time of laparoscopy, incubated overnight to generate single-cell suspensions, and frozen for later scRNA-seq processing. The scRNA-seq libraries were prepared using the Chromium Single-Cell 3′ Reagent Kit v2.0 (10x Genomics) and were subsequently sequenced using the Illumina HiSeq 4000 (Jussi Taipale Lab at the Karolinska Institutet, Sweden) as well as the HiSeq 2500 and NovaSeq 6000 (Sequencing Unit of the Institute for Molecular Medicine Finland). The fastq files were processed using the Cell Ranger software v.6.0.1, including steps for demultiplexing, alignment, barcode filtering, and unique molecular identifier (UMI) quantification. The reference index was created using the GRCh38.d1.vd1 genome with GENCODE v25 annotation. The filtered feature-barcode matrices were pre-processed using the Seurat v4.0.1 toolkit.^75^ Cells having more than 20% of UMI counts originating from mitochondrial genes were filtered out.

##### Cell type annotation

Cell type annotation was performed on a broader cohort of 95 single-cell samples^14^^,^^19^^,^^24^^,^^76^^,^^77^ (Figures S5A and S5B). The major cell types were identified using a KNN graph-based clustering algorithm and canonical cell type markers: epithelial cancer cells (*MUC16*, *PAX8*, *WFDC2*), stromal cells (*COL1A2*, *DCN*, *FGFR1*, *VIM*), and immune cells (*CD14*, *CD3D*, *CD79B*, *CD8A*, *FCER1G*, *HLA-DRA*, *NKG7*, *PTPRC*). The QC thresholds were determined for each cell type separately, and the cells having log2(counts) smaller than the threshold were removed. The threshold was 12.5 for epithelial cancer cells, 10 for immune cells, and 11 for stromal cells. Potential doublets were filtered utilizing the DoubletFinder v2.0.4 R package.^69^ After ensuring the high quality of the cells, the cells were clustered again following the standard Seurat workflow. Cells were assigned to higher resolution cell types utilizing markers used in a previous publication.^78^ Consistency of the final annotation was ensured by cross-checking the results with canonical markers (Figure S4A).

##### IFN-I and TFs activity score assessment

To assess the IFN-I pathway activity, we employed the Python implementation of the DecoupleR^71^ framework v1.6.0 and retrieved the Hallmark gene sets from MSigDB^23^ with *decoupler.get_resource(“MSigDB”)*. For the inference of enrichment scores of the IFN-I response pathway, we ran the over-representation analysis (ORA) executed via *decoupler.run_ora()*. To analyze the IFN-I enrichment scores, we scaled the Hallmark Interferon Alpha Response scores to range between 0 and 1 and visualized this data as boxplots (Figure 4A). Additionally, stacked bar plots were overlaid to show the proportion of cells falling within predefined ranges of IFN-I response score. These were visualized alongside the boxplot for each tumor sample. To obtain transcription factor activities, we retrieved the CollecTRI^21^ gene regulatory network with *decoupler.get_collectri()* and utilized the univariate linear model method executed via *decoupler.run_ulm()*.

##### Cellular states assignment

Cellular states were annotated by performing ORA using the marker sets curated in our previous publication.^14^ For each malignant cell, states were ranked based on the enrichment scores executed via *decoupler.run_ora()*, and the top-ranked cellular state was assigned.

#### WGS data analysis

##### WGS data preprocessing

The genomic DNA data processing was performed with the Anduril2 workflow platform^45^ and included quality control, alignment to the reference human genome, deduplication, cross-sample contamination estimation, and variant discovery. We used FastQC^47^ v0.11.4 and Trimmomatic^46^ v0.32 for sequenced DNA read quality control and trimming steps. Subsequently, the high-quality reads underwent alignment to the reference human genome GRCh38.d1.vd1 using BWA-MEM^48^ v0.7.12-r1039 with default parameters, subjected to deduplication with Picard v2.6 (https://broadinstitute.github.io/picard/) and base quality recalibration using the Genome Analysis Toolkit^49^ (GATK) v4.1.9.0. Additionally, cross-sample contamination estimation was conducted with GATK, setting the contamination estimation threshold at 3% for tumors, 5% for normals in the Panel of Normals (PoN), and 10% for normals in germline variant calling.

##### Mutation calling

Somatic short variants were identified by collectively analyzing multiple tumor samples against a single matched normal sample for each patient. This analysis was performed using GATK v4.1.9.0 Mutect2,^79^ following established best practices.^80^ The Finnish gnomAD^81^ v3.0 allele frequencies served as the germline resource for variant calling. We used a PoN generated with 181 normal samples from the DECIDER study and 99 TCGA normals.^7^ Afterward, the GATK FilterMutectCalls tool was employed for variant filtration, retaining only those variants that successfully passed all applied filters. Variant allele frequencies (VAF) were computed by considering the read depths for both reference and alternate alleles as indicated in the AD field. We annotated the variants using the GATK VariantAnnotator, adding dbSNP^82^ 155 IDs. Additionally, an offline version of Combined Annotation Dependent Depletion^54^ (CADD) v1.6, complemented by an in-house solution, was employed to annotate variant call format files. The annotation process was further enriched using ANNOVAR^55^ 20191024, adding refGene^83^ data dated 2020-08-16, together with other annotations adapted for ANNOVAR, including ClinVar^84^ 20220816, customized annotations for COSMIC^85^ v96, and gnomAD v3.0 genomes.

Germline variants were called as previously described.^7^ The allele depths of variants at exons, untranslated regions, and splice sites were estimated by forced calling using GATK 4.1.9.0 Mutect2 in joint calling mode, as in somatic variant calling.

##### Tumor fraction estimation

Tumor fraction was estimated using a modified ASCAT^50^ algorithm that inputs copy-number segmentation from GATK, as previously described.^18^

##### Copy number and breakpoint calling

Breakpoint and copy-number segmentation were determined by employing a combination of GRIDSS^61^ and the Hartwig Medical Foundation (HMF) toolkit. The pipeline was constructed on the Nextflow^86^ platform, utilizing default settings for the incorporated tools. Breakpoint calling was executed using GRIDSS^61^ v2.13.2, with the exclusion of regions found in both the ENCODE^87^ and in-house built DECIDER blacklists.^7^ Subsequently, breakpoint filtering was accomplished through GRIPSS^62^ v2.0, leveraging a PoN originating from blood samples collected from DECIDER patients and a Dutch population. B allele frequency (BAF) calculation was conducted using the Amber tool v3.8, while heterozygous biallelic loci were identified as described in the preceding section. Cobalt v1.12 was employed to assess read depth normalized for GC content. The combined results from all these tools, in addition to somatic single-nucleotide variants (SNVs), were then used to input data into PURPLE^62^^,^^88^ v3.7.2. PURPLE estimated copy-number segmentation profiles and conducted estimations for tumor purity and ploidy of the samples. Breakpoints derived from GRIDSS-PURPLE analysis were used for structural variants annotation with Linx^89^ v1.22. Among all events, simple and chained foldback inversions were extracted and used for the analysis.

##### Mutational signatures and signature events

Mutational signatures were fitted, and SBS3- and ID6-based homologous recombination deficiency (HRD) statuses were computed as previously described^90^ using COSMIC^91^ v3.3.1 reference signatures. A cancer was classified as positive for foldback inversions if at least one tissue sample was positive. A sample sequenced with NovaSeq was called positive if at least five foldback inversion events were detected, and a sample sequenced with BGISEQ or HiSeq was called positive if at least three foldback inversion events were detected to accommodate the platform-specific differences in detection sensitivity.

##### Curation of somatic aberrations

To define the genomic status of genes involved in the IFN-I signaling cascade, we curated function-affecting mutations and the dosage of intact gene copies and summarized the dosage perturbation over the whole signaling cascade. First, we collected short somatic mutations and annotated, weighing a mutation as a loss-of-function if its consequence was protein truncation or if it was a missense mutation predicted deleterious unanimously by PolyPhen2^63^ and SIFT.^92^ Then, we retrieved all genomic breaks from the PURPLE-processed data and assessed their effect on the coding sequence. With locus copy number and tumor fraction in the sample from the processed data and with read counts supporting the aberration, we estimated the number of gene copies affected by the short or structural aberrations and the number of intact gene copies. Using the most common copy number across the cancer genome as a reference copy number, we calculated the dosage of intact gene copies as a ratio of the number of intact gene copies and the reference copy number. Gene dosage below or equal to 0.5 or above or equal to 2.0 was considered perturbed. Adapting the molecular distance introduced earlier,^93^ a pathway perturbation score of cancer was calculated as a log2-scale sum of absolute perturbed gene dosages.

#### Tissue cyclic multiplex immunofluorescence (t-CycIF)

Whole-slide HGSC FFPE samples were stained and scanned iteratively with validated antibodies (DAPI, E-Cadherin (CST, CAT 3199), CK7 (Abcam, CAT ab209601), Vimentin (CST, CAT 9855), pSTAT1 (CST, CAT 8183S)) using RareCyte CyteFinder scanner following the t-CycIF workflow^94^ as described.^24^ Briefly, immuno-staining with antibodies was followed by DAPI staining to mark for nuclei to enable subsequent image analysis. BaSiC tool and the Ashlar algorithm were used for image correcting, stitching, and registration.^65^ Single-cell nuclei segmentation was performed using probability maps created by UNET,^66^ and these were dilated by 2px to obtain whole-cell segmentation masks. The mean fluorescence intensity for each cell was computed using in-house scripts (https://github.com/farkkilab/image_processing) and Python’s scikit-learn library to obtain a single-cell data table. SOM-based TRIBUS^25^ algorithm was used for cell type calling, and pSTAT1 expression was gated and rescaled (*pl.gate_finder* and *pp.rescale* using the scimap package in Python) to a value between 0 and 1 separately for each image so that values above 0.5 identify cells expressing the marker as described.^24^

#### Functional cell lines experiments

##### Cell viability assay

To assess the relative 50% inhibitory concentration (IC50) of COV362 or Kuramochi cells to cisplatin and IFN-alpha, cell viability assays were performed. Briefly, cells (5,000 cells/well) were seeded in 96-well plates and cultured overnight, then treated with either vehicle or different concentrations of cisplatin (0.5, 1, 2.5, 5, 10, 20, 30, 50μM) or IFN-alpha (0.01, 0.1, 1, 10, 100, 1000U/μl) for 72 h. For combination treatment, cells were treated with vehicle or 10U/μl of IFN-alpha combined with increasing concentration of cisplatin (0.5, 1, 2.5, 5, 10, 20, 30, 50μM) for 72h. Cell viability was assessed by measuring luminescence using CellTiter-Glo 2.0 Cell Viability Assay (Promega) in 2014 Envision multilabel reader (PerkinElmer). Dose-response curves and relative IC50 were generated by GraphPad Prism 10. The IC50 curves of cisplatin for COV362 and Kuramochi cells are shown in Figure S6, and the IC50 curves of IFN-alpha for COV362 and Kuramochi cells are shown in Figure S6G.

##### Antibody-oligo conjugation

Oligonucleotides were conjugated to two antibodies against HGSOC cell surface proteins β2-Microglobulin (β2M) and CD298 (BioLegend) by iEDDA-click chemistry according to CITE-seq antibody-oligo conjugation protocol (https://cite-seq.com). Briefly, oligonucleotides were derivatized with TCO-PEG4-NHS (Click chemistry tools) in 10x borate buffered saline (BBS), and the TCO-labeled oligo was verified by Bioanalyzer Small RNA chip (Agilent). Antibodies were conjugated to mTz-PEG4-NHS (Click chemistry tools) at first in 1x BBS, then a 300μL reaction containing 300μg of mTz-PEG4-antibodies and 3nmol of TCO-PEG4-Oligo in 1x BBS was reacted at 4°C overnight. Excess mTz was quenched with 30μL of 10mM TCO-PEG4-glycine at room temperature for 10 min. Excess oligos were washed by Amicon Ultra-0.5 centrifugal filter unit with 50kDa MWCO membrane (Millipore) with 1x PBS. The conjugation efficiency was assessed by size shift of the conjugated antibodies on a 4–12% PAGE-gel (Thermo Fisher Scientific).

##### Single-cell RNA seq combined with cell hashing

This experiment was performed using Chromium Next GEM Single Cell 3′ Reagent Kits v3.1 (Dual Index) (10x Genomics, CG000317 Rev C). COV362 or Kuramochi cells were seeded in the 96-well plate, cultured overnight, and treated with cisplatin at different doses (10%, 50%, and 200% of IC50) and for different treatment times (24h, 12h). Subsequently, cells in different treatment conditions were labeled with distinct oligo-conjugated antibodies (Hashtags) against HGSC cell surface proteins β2-Microglobulin (β2M) and CD298. All cells were pooled and subjected to one 10x single-cell RNA seq run. The gene expression and hashtag libraries for NGS were constructed and sequenced according to the manufacturer’s protocol. Library quality was assessed on the Bioanalyser using the Agilent High Sensitivity DNA kit (Agilent). Libraries were sequenced on the Illumina NextSeq 2000 sequencing platform according to 10x manufacturer’s protocol. Raw base call files were demultiplexed into FASTQ files, aligned to the GRCh38 human reference genome, filtered, and processed to count barcodes and UMIs using the Cell Ranger software (v6.0.2, 10x Genomics). Cell demultiplexing was performed using the demultiplexing function HTODemux from the R package Seurat v4.2.0. The subsequent analysis used the single-cell filtered count matrix of all the genes.

##### Single-cell RNA-seq analysis for cell line experiments

We performed five replicated experiments for each cell line (COV362 and Kuramochi) under different concentrations of cisplatin (10%, 50%, and 200%) and at two time points (12h and 24h). The following steps were taken to analyze the data.

##### Gene ranking based on cisplatin-induced expression changes

For each experiment (*n* = 10), we calculated the effect size (Cohen’s *d*) between cisplatin-treated cells (10%, 50%, and 200%; 12h and 24h) and control cells (control and vehicle) for each gene. This produced a vector of effect sizes per gene, ranking them from upregulated to downregulated due to cisplatin exposure. We treated this vector as the “cisplatin gene expression signature.” To evaluate the agreement between the 10 gene expression signatures, Pearson correlation coefficients were calculated. The universal cisplatin gene expression signature was obtained by averaging the effect sizes across the 10 experiments for each gene.

##### Classification of treated cells by cisplatin sensitivity

For cells treated with 200% cisplatin for 24h, we calculated a cisplatin sensitivity score (CisSenScore). This was computed as the log ratio between counts in the 1,000 genes with the highest effect sizes (based on the universal cisplatin gene expression signature described above) and the 10,000 genes with the lowest effect sizes (to control for total count variations). To ensure robustness, we tested additional thresholds for defining cisplatin sensitivity (top 1%, 5%, and 10% genes vs. the rest), yielding consistent results (Figure S7). For each experiment (*n* = 10), we classified treated cells (200%, 24h) into ‘more-sensitive’ (top 25% of CisSenScores) and ‘less-sensitive’ (bottom 25%).

##### Transcriptional programs of heterogeneity in control cells

For each experiment (*n* = 10), we performed principal component analysis (PCA) on the centered gene expression values from control cells (no cisplatin treatment). This captured the underlying heterogeneity in gene expression prior to treatment. We selected the first 10 principal components (PCs) from each experiment, as these captured most of the variance. Given the replication of the experiments (5 per cell line), we expected similar patterns across experiments. By assessing the agreement between replicates, we verified the robustness of the identified patterns.

##### Identification of transcriptional programs linked to cisplatin response

For each experiment (*n* = 10), we projected the gene expression values of treated cells onto the PC space derived from control cells (from the PCA performed as described above). The PCA was calculated using the *prcomp()* function in R, and the projection of treated cells into the control PC space was performed with the *predict()* function.

For each PC, we calculated Cohen’s *d* (using the *cohen.d()* function from the effsize R package) to quantify the effect size between ‘more-sensitive’ and ‘less-sensitive’ cells classified as described above. To control for potential cell cycle effects, we also calculated Cohen’s *d* between the cell cycle phases (G1 vs. S, G1 vs. G2/M, and S vs. G2/M) for each PC. PCs that separated sensitivity groups (Cohen’s *d* > 0.5) but did not separate cell cycle phases (Cohen’s *d* < 0.5 for all phases) were selected for further analysis.

We identified 27 PCs (out of 100) that met these criteria, representing transcriptional programs in control cells that were associated with differential cisplatin sensitivity when upregulated or downregulated in treated cells.

##### Characterization of transcriptional programs linked to cisplatin sensitivity

Since we had 5 replicates per cell line, we expected to find the same transcriptional programs across experiments. To validate these patterns, we focused on transcriptional programs that consistently appeared across replicates. We performed hierarchical clustering of the 27 selected PCs and identified three clusters representing robust transcriptional programs associated with cisplatin sensitivity.

For each cluster, we calculated the mean transcriptional program across all PCs within the cluster by gene. To further characterize these programs, we used GSEA^22^ to assess pathway activities. Genes were ranked by the median gene weight across signatures within each cluster, and GSEA^22^ was performed using the Hallmark gene sets from the MSigDB^23^ collection (ClusterProfiler package).

### Quantification and statistical analysis

All statistical analyses were performed in R v4.5.0, Python v3.8.10, and Prism (GraphPad). Statistical tests for patient and cancer characteristics were performed using R with the Fisher exact test for categorical variables and the Mann-Whitney U-test for ordinal or non-normal continuous variables. Statistical tests for transcription factor and pathway activities in bulk RNA-seq were performed using Python (package decoupleR v1.6.0), using a gene-weighted univariate linear model for CollecTRI TFs and a multivariate linear model for PROGENy pathways, with two-sided t-statistics references to the appropriate t-distribution to obtain raw *p* values, which were further adjusted with Benjamini–Hochberg (BH) FDR; the resulting FDR-adjusted *p* values are reported throughout. For scRNA-seq comparisons between chemo-refractory and chemo-sensitive groups, we employed a Generalized Estimating Equations (GEE) regression model (Python package statsmodels v0.14.1) to account for patient-level variability. Permutation tests (*n* = 10,000) were used to evaluate the statistical significance of differences in pathway-activity scores at the patient level, and Levene’s test was applied to assess variance differences across groups. The strength of correlations was measured using the Spearman (ρ) correlation coefficient with corresponding two-sided *p* values. The threshold for significance was set at *p* ≤ 0.05 (adjusted or raw) in all analyses unless specified otherwise. The BH FDR procedure was used to adjust the two-sided *p* values for multiple comparisons whenever more than one comparison was made.

Patient survival was analyzed using R (library survival v3.8.3), with a diagnosis of HGSC as the starting point of time at risk and death from HGSC as the endpoint. The follow-up was cut at 2023-01-31, and patients were censored at that time, if alive. One patient was censored at the time of non-HGSC-related death. Hazard ratios were estimated for the PROGENy JAK-STAT *Z* score in the solid metastatic or intra-abdominal sample with the lowest JAK-STAT score at diagnosis with a multivariable Cox model adjusted for HRD status from SBS3 mutational signature, presence of pathogenic mutations in homologous recombination (HR) genes, high volume (>1000 mL) of ascites at diagnosis and presence of macroscopic residual tumor after cytoreductive surgery as dichotomous variables, and, cancer dissemination score^16^ as a linear variable. For visualization with Kaplan-Meier survival estimator curves, both the NACT-treated subcohort (*n* = 82 patients with bulk RNA-seq data) and the TCGA cohort (*n* = 303 patients with bulk RNA-seq data) were separately classified into three equally sized groups based on the JAK-STAT scores of corresponding bulk RNA-seq samples. The separation of the curves was measured with a log rank test (high tertile vs. low tertile).

Statistical testing of IFN-alpha and cisplatin effect on cell viability was performed in GraphPad Prism 10 using the nonlinear regression and F-test to compare the curve fit from different treatment models.

Statistical tests related to figures are indicated in the figure legends. The number of individuals or samples in each test is explained in the Results and/or figure captions. Further statistical details of the analyses are defined in the corresponding method sections of the STAR Methods.

### Additional resources

This study is a part of the DECIDER trial, ClinicalTrials.gov with identifier: NCT04846933, accessed at https://clinicaltrials.gov/ct2/show/NCT04846933.

The visualization of genomic data was performed using GenomeSpy and is available at https://csbi.ltdk.helsinki.fi/p/chemorefractory/.

## Supplemental information

Document S1. Figures S1–S8 and Tables S1, S2, S4, and S5

Table S3. Availability of clinical data, bulk RNA-seq, WGS, scRNA-seq, and CycIF samples from diagnostic biopsies of 31 patients with chemo-refractory HGSC and 62 patients with chemo-sensitive HGSC that were included in this study

Data S1. Pathway activity scores for JAK-STAT and hypoxia pathways computed with PROGENy for 58 bulk RNA-seq samples from patients with HGSC in the discovery cohort

Data S2. Estimated cell type proportions for each bulk RNA-seq sample derived using the PRISM deconvolution method

Data S3. Estimated cell type proportions for each bulk RNA-seq sample derived using the Kassandra deconvolution method

Document S2. Article plus supplemental information

## References

[1] Cabasag C.J., Fagan P.J., Ferlay J., Vignat J., Laversanne M., Liu L., van der Aa M.A., Bray F., Soerjomataram I. (2022). Ovarian cancer today and tomorrow: A global assessment by world region and Human Development Index using GLOBOCAN 2020. Int. J. Cancer.

[2] Latifi A., Luwor R.B., Bilandzic M., Nazaretian S., Stenvers K., Pyman J., Zhu H., Thompson E.W., Quinn M.A., Findlay J.K., Ahmed N. (2012). Isolation and Characterization of Tumor Cells from the Ascites of Ovarian Cancer Patients: Molecular Phenotype of Chemoresistant Ovarian Tumors. PLoS One.

[3] Labidi-Galy S.I., Papp E., Hallberg D., Niknafs N., Adleff V., Noe M., Bhattacharya R., Novak M., Jones S., Phallen J. (2017). High grade serous ovarian carcinomas originate in the fallopian tube. Nat. Commun..

[4] Doubeni C.A., Doubeni A.R., Myers A.E. (2016). Diagnosis and Management of Ovarian Cancer. Am. Fam. Physician.

[5] Jamalzadeh S., Dai J., Lavikka K., Li Y., Jiang J., Huhtinen K., Virtanen A., Oikkonen J., Hietanen S., Hynninen J. (2024). Genome-wide quantification of copy-number aberration impact on gene expression in ovarian high-grade serous carcinoma. BMC Cancer.

[6] Cancer Genome Atlas Research Network (2011). Integrated genomic analyses of ovarian carcinoma. Nature.

[7] Lahtinen A., Lavikka K., Virtanen A., Li Y., Jamalzadeh S., Skorda A., Lauridsen A.R., Zhang K., Marchi G., Isoviita V.-M. (2023). Evolutionary states and trajectories characterized by distinct pathways stratify patients with ovarian high grade serous carcinoma. Cancer Cell.

[8] Torre L.A., Trabert B., DeSantis C.E., Miller K.D., Samimi G., Runowicz C.D., Gaudet M.M., Jemal A., Siegel R.L. (2018). Ovarian cancer statistics, 2018. CA Cancer J. Clin..

[9] SEER∗Explorer (2024).

[10] González-Martín A., Harter P., Leary A., Lorusso D., Miller R.E., Pothuri B., Ray-Coquard I., Tan D.S.P., Bellet E., Oaknin A., Ledermann J. (2023). Newly diagnosed and relapsed epithelial ovarian cancer: ESMO Clinical Practice Guideline for diagnosis, treatment and follow-up. Ann. Oncol..

[11] Ray-Coquard I., Leary A., Pignata S., Cropet C., González-Martín A., Marth C., Nagao S., Vergote I., Colombo N., Mäenpää J. (2023). Olaparib plus bevacizumab first-line maintenance in ovarian cancer: final overall survival results from the PAOLA-1/ENGOT-ov25 trial. Ann. Oncol..

[12] Matsuo K., Matsuzaki S., Nusbaum D.J., Maoz A., Oda K., Klar M., Roman L.D., Sood A.K. (2021). Possible candidate population for neoadjuvant chemotherapy in women with advanced ovarian cancer. Gynecol. Oncol..

[13] Ledermann J.A., Raja F.A., Fotopoulou C., Gonzalez-Martin A., Colombo N., Sessa C., ESMO Guidelines Working Group (2013). Newly diagnosed and relapsed epithelial ovarian carcinoma: ESMO Clinical Practice Guidelines for diagnosis, treatment and follow-up. Ann. Oncol..

[14] Zhang K., Erkan E.P., Jamalzadeh S., Dai J., Andersson N., Kaipio K., Lamminen T., Mansuri N., Huhtinen K., Carpén O. (2022). Longitudinal single-cell RNA-seq analysis reveals stress-promoted chemoresistance in metastatic ovarian cancer. Sci. Adv..

[15] Eisenhauer E.A., Therasse P., Bogaerts J., Schwartz L.H., Sargent D., Ford R., Dancey J., Arbuck S., Gwyther S., Mooney M. (2009). New response evaluation criteria in solid tumours: Revised RECIST guideline (version 1.1). Eur. J. Cancer.

[16] Isoviita V.-M., Salminen L., Azar J., Lehtonen R., Roering P., Carpén O., Hietanen S., Grénman S., Hynninen J., Färkkilä A., Hautaniemi S. (2019). Open Source Infrastructure for Health Care Data Integration and Machine Learning Analyses. JCO Clin. Cancer Inform..

[17] Beroukhim R., Getz G., Nghiemphu L., Barretina J., Hsueh T., Linhart D., Vivanco I., Lee J.C., Huang J.H., Alexander S. (2007). Assessing the significance of chromosomal aberrations in cancer: Methodology and application to glioma. Proc. Natl. Acad. Sci. USA.

[18] Lavikka K., Oikkonen J., Li Y., Muranen T., Micoli G., Marchi G., Lahtinen A., Huhtinen K., Lehtonen R., Hietanen S. (2024). Deciphering cancer genomes with GenomeSpy: a grammar-based visualization toolkit. GigaScience.

[19] Häkkinen A., Zhang K., Alkodsi A., Andersson N., Erkan E.P., Dai J., Kaipio K., Lamminen T., Mansuri N., Huhtinen K. (2021). PRISM: recovering cell-type-specific expression profiles from individual composite RNA-seq samples. Bioinformatics.

[20] Schubert M., Klinger B., Klünemann M., Sieber A., Uhlitz F., Sauer S., Garnett M.J., Blüthgen N., Saez-Rodriguez J. (2018). Perturbation-response genes reveal signaling footprints in cancer gene expression. Nat. Commun..

[21] Müller-Dott S., Tsirvouli E., Vazquez M., Ramirez Flores R.O., Badia-I-Mompel P., Fallegger R., Türei D., Lægreid A., Saez-Rodriguez J. (2023). Expanding the coverage of regulons from high-confidence prior knowledge for accurate estimation of transcription factor activities. Nucleic Acids Res..

[22] Subramanian A., Tamayo P., Mootha V.K., Mukherjee S., Ebert B.L., Gillette M.A., Paulovich A., Pomeroy S.L., Golub T.R., Lander E.S., Mesirov J.P. (2005). Gene set enrichment analysis: a knowledge-based approach for interpreting genome-wide expression profiles. Proc. Natl. Acad. Sci. USA.

[23] Liberzon A., Subramanian A., Pinchback R., Thorvaldsdóttir H., Tamayo P., Mesirov J.P. (2011). Molecular signatures database (MSigDB) 3.0. Bioinformatics.

[24] Launonen I.-M., Niemiec I., Hincapié-Otero M., Erkan E.P., Junquera A., Afenteva D., Falco M.M., Liang Z., Salko M., Chamchougia F. (2024). Chemotherapy induces myeloid-driven spatially confined T cell exhaustion in ovarian cancer. Cancer Cell.

[25] Kang Z., Szabo A., Farago T., Perez-Villatoro F., Junquera A., Shah S., Launonen I.M., Anttila E., Casado J., Färkkilä A. (2024). Tribus: Semi-automated discovery of cell identities and phenotypes from multiplexed imaging and proteomic data. Bioinformatics).

[26] Domcke S., Sinha R., Levine D.A., Sander C., Schultz N. (2013). Evaluating cell lines as tumour models by comparison of genomic profiles. Nat. Commun..

[27] Haley J., Tomar S., Pulliam N., Xiong S., Perkins S.M., Karpf A.R., Mitra S., Nephew K.P., Mitra A.K. (2016). Functional characterization of a panel of high-grade serous ovarian cancer cell lines as representative experimental models of the disease. Oncotarget.

[28] Kim C., Gao R., Sei E., Brandt R., Hartman J., Hatschek T., Crosetto N., Foukakis T., Navin N.E. (2018). Chemoresistance Evolution in Triple-Negative Breast Cancer Delineated by Single-Cell Sequencing. Cell.

[29] França G.S., Baron M., King B.R., Bossowski J.P., Bjornberg A., Pour M., Rao A., Patel A.S., Misirlioglu S., Barkley D. (2024). Cellular adaptation to cancer therapy along a resistance continuum. Nature.

[30] Chowdhury S., Kennedy J.J., Ivey R.G., Murillo O.D., Hosseini N., Song X., Petralia F., Calinawan A., Savage S.R., Berry A.B. (2023). Proteogenomic analysis of chemo-refractory high-grade serous ovarian cancer. Cell.

[31] Hanrahan A.J., Chen Z., Rosen N., Solit D.B. (2024). BRAF — a tumour-agnostic drug target with lineage-specific dependencies. Nat. Rev. Clin. Oncol..

[32] Havasi A., Cainap S.S., Havasi A.T., Cainap C. (2023). Ovarian Cancer-Insights into Platinum Resistance and Overcoming It. Medicina (Kaunas).

[33] Li H., Lin R., Zhang Y., Zhu Y., Huang S., Lan J., Lu N., Xie C., He S., Zhang W. (2024). N6-methyladenosine-modified circPLPP4 sustains cisplatin resistance in ovarian cancer cells via PIK3R1 upregulation. Mol. Cancer.

[34] Li J., Pan C., Boese A.C., Kang J., Umano A.D., Magliocca K.R., Yang W., Zhang Y., Lonial S., Jin L., Kang S. (2020). DGKA Provides Platinum Resistance in Ovarian Cancer Through Activation of c-JUN-WEE1 Signaling. Clin. Cancer Res..

[35] Silva R., Glennon K., Metoudi M., Moran B., Salta S., Slattery K., Treacy A., Martin T., Shaw J., Doran P. (2023). Unveiling the epigenomic mechanisms of acquired platinum-resistance in high-grade serous ovarian cancer. Int. J. Cancer.

[36] Zhu Y., Liang L., Zhao Y., Li J., Zeng J., Yuan Y., Li N., Wu L. (2024). CircNUP50 is a novel therapeutic target that promotes cisplatin resistance in ovarian cancer by modulating p53 ubiquitination. J. Nanobiotechnology.

[37] Bowman A., Fergusson R.J., Allan S.G., Stewart M.E., Gregor A., Cornbleet M.A., Greening A.P., Crompton G.K., Leonard R.C., Smyth J.F. (1990). Potentiation of cisplatin by alpha-interferon in advanced non-small cell lung cancer (NSCLC): a phase II study. Ann. Oncol..

[38] Salvagno C., Ciampricotti M., Tuit S., Hau C.-S., van Weverwijk A., Coffelt S.B., Kersten K., Vrijland K., Kos K., Ulas T. (2019). Therapeutic targeting of macrophages enhances chemotherapy efficacy by unleashing type I interferon response. Nat. Cell Biol..

[39] Marks Z.R.C., Campbell N.K., Mangan N.E., Vandenberg C.J., Gearing L.J., Matthews A.Y., Gould J.A., Tate M.D., Wray-McCann G., Ying L. (2023). Interferon-ε is a tumour suppressor and restricts ovarian cancer. Nature.

[40] Kureshi C.T., Dougan S.K. (2025). Cytokines in cancer. Cancer Cell.

[41] Berek J.S. (2000). Interferon plus chemotherapy for primary treatment of ovarian cancer. Lancet.

[42] Bezwoda W.R., Golombick T., Dansey R., Keeping J. (1991). Treatment of malignant ascites due to recurrent/refractory ovarian cancer: the use of interferon-alpha or interferon-alpha plus chemotherapy in vivo and in vitro. Eur. J. Cancer.

[43] Berek J.S., Welander C., Schink J.C., Grossberg H., Montz F.J., Zigelboim J. (1991). A phase I-II trial of intraperitoneal cisplatin and alpha-interferon in patients with persistent epithelial ovarian cancer. Gynecol. Oncol..

[44] Hall G.D., Brown J.M., Coleman R.E., Stead M., Metcalf K.S., Peel K.R., Poole C., Crawford M., Hancock B., Selby P.J., Perren T.J. (2004). Maintenance treatment with interferon for advanced ovarian cancer: results of the Northern and Yorkshire gynaecology group randomised phase III study. Br. J. Cancer.

[45] Cervera A., Rantanen V., Ovaska K., Laakso M., Nuñez-Fontarnau J., Alkodsi A., Casado J., Facciotto C., Häkkinen A., Louhimo R. (2019). Anduril 2: upgraded large-scale data integration framework. Bioinformatics.

[46] Bolger A.M., Lohse M., Usadel B. (2014). Trimmomatic: a flexible trimmer for Illumina sequence data. Bioinformatics.

[47] Andrews S. (2010). FASTQC. A quality control tool for high throughput sequence data. https://www.bioinformatics.babraham.ac.uk/projects/fastqc/.

[48] Li H. (2013). Aligning sequence reads, clone sequences and assembly contigs with BWA-MEM. arXiv.

[49] McKenna A., Hanna M., Banks E., Sivachenko A., Cibulskis K., Kernytsky A., Garimella K., Altshuler D., Gabriel S., Daly M., DePristo M.A. (2010). The Genome Analysis Toolkit: A MapReduce framework for analyzing next-generation DNA sequencing data. Genome Res..

[50] Van Loo P., Nordgard S.H., Lingjærde O.C., Russnes H.G., Rye I.H., Sun W., Weigman V.J., Marynen P., Zetterberg A., Naume B. (2010). Allele-specific copy number analysis of tumors. Proc. Natl. Acad. Sci. USA.

[51] Quinlan A.R., Hall I.M. (2010). BEDTools: a flexible suite of utilities for comparing genomic features. Bioinformatics.

[52] Quinlan A.R. (2014). BEDTools: The Swiss-Army Tool for Genome Feature Analysis. Curr. Protoc. Bioinformatics.

[53] Mermel C.H., Schumacher S.E., Hill B., Meyerson M.L., Beroukhim R., Getz G. (2011). GISTIC2.0 facilitates sensitive and confident localization of the targets of focal somatic copy-number alteration in human cancers. Genome Biol..

[54] Rentzsch P., Schubach M., Shendure J., Kircher M. (2021). CADD-Splice-improving genome-wide variant effect prediction using deep learning-derived splice scores. Genome Med..

[55] Wang K., Li M., Hakonarson H. (2010). ANNOVAR: functional annotation of genetic variants from high-throughput sequencing data. Nucleic Acids Res..

[56] Icay K., Chen P., Cervera A., Rantanen V., Lehtonen R., Hautaniemi S. (2016). SePIA: RNA and small RNA sequence processing, integration, and analysis. BioData Min..

[57] Dobin A., Davis C.A., Schlesinger F., Drenkow J., Zaleski C., Jha S., Batut P., Chaisson M., Gingeras T.R. (2013). STAR: ultrafast universal RNA-seq aligner. Bioinformatics.

[58] Roberts A., Pachter L. (2013). Streaming fragment assignment for real-time analysis of sequencing experiments. Nat. Methods.

[59] Holmström S., Hautaniemi S., Häkkinen A. (2022). POIBM: batch correction of heterogeneous RNA-seq datasets through latent sample matching. Bioinformatics.

[60] Zaitsev A., Chelushkin M., Dyikanov D., Cheremushkin I., Shpak B., Nomie K., Zyrin V., Nuzhdina E., Lozinsky Y., Zotova A. (2022). Precise reconstruction of the TME using bulk RNA-seq and a machine learning algorithm trained on artificial transcriptomes. Cancer Cell.

[61] Cameron D.L., Baber J., Shale C., Valle-Inclan J.E., Besselink N., Van Hoeck A., Janssen R., Cuppen E., Priestley P., Papenfuss A.T. (2021). GRIDSS2: comprehensive characterisation of somatic structural variation using single breakend variants and structural variant phasing. Genome Biol..

[62] Cameron D.L., Baber J., Shale C., Papenfuss A.T., Valle-Inclan J.E., Besselink N., Cuppen E., Priestley P. (2019). GRIDSS, PURPLE, LINX: Unscrambling the tumor genome via integrated analysis of structural variation and copy number. Bioinformatics.

[63] Adzhubei I.A., Schmidt S., Peshkin L., Ramensky V.E., Gerasimova A., Bork P., Kondrashov A.S., Sunyaev S.R. (2010). A method and server for predicting damaging missense mutations. Nat. Methods.

[64] Ng P.C., Henikoff S. (2001). Predicting Deleterious Amino Acid Substitutions. Genome Res..

[65] Muhlich J.L., Chen Y.-A., Yapp C., Russell D., Santagata S., Sorger P.K. (2022). Stitching and registering highly multiplexed whole-slide images of tissues and tumors using ASHLAR. Bioinformatics.

[66] Ronneberger O., Fischer P., Brox T., Navab N., Hornegger J., Wells W.M., Frangi A.F. (2015). Medical Image Computing and Computer-Assisted Intervention – MICCAI 2015 Lecture Notes in Computer Science.

[67] Gu Z. (2022). Complex heatmap visualization. Imeta.

[68] Butler A., Hoffman P., Smibert P., Papalexi E., Satija R. (2018). Integrating single-cell transcriptomic data across different conditions, technologies, and species. Nat. Biotechnol..

[69] McGinnis C.S., Murrow L.M., Gartner Z.J. (2019). DoubletFinder: Doublet Detection in Single-Cell RNA Sequencing Data Using Artificial Nearest Neighbors. Cell Syst..

[70] Wolf F.A., Angerer P., Theis F.J. (2018). SCANPY: large-scale single-cell gene expression data analysis. Genome Biol..

[71] Badia-I-Mompel P., Vélez Santiago J., Braunger J., Geiss C., Dimitrov D., Müller-Dott S., Taus P., Dugourd A., Holland C.H., Ramirez Flores R.O., Saez-Rodriguez J. (2022). decoupleR: ensemble of computational methods to infer biological activities from omics data. Bioinform. Adv..

[72] Muzellec B., Teleńczuk M., Cabeli V., Andreux M. (2023). PyDESeq2: a python package for bulk RNA-seq differential expression analysis. Bioinformatics.

[73] Fang Z., Liu X., Peltz G. (2023). GSEApy: a comprehensive package for performing gene set enrichment analysis in Python. Bioinformatics.

[74] Nirmal A.J., Sorger P.K. (2024). SCIMAP: A Python Toolkit for Integrated Spatial Analysis of Multiplexed Imaging Data. J. Open Source Softw..

[75] Hao Y., Hao S., Andersen-Nissen E., Mauck W.M., Zheng S., Butler A., Lee M.J., Wilk A.J., Darby C., Zager M. (2021). Integrated analysis of multimodal single-cell data. Cell.

[76] Hippen A.A., Falco M.M., Weber L.M., Erkan E.P., Zhang K., Doherty J.A., Vähärautio A., Greene C.S., Hicks S.C. (2021). miQC: An adaptive probabilistic framework for quality control of single-cell RNA-sequencing data. PLoS Comput. Biol..

[77] Pirttikoski A., Gall-Mas L., Senkowski W., Fontaneda-Arenas D., Marin Falco M., Erkan E.P., Hynninen J., Wennerberg K., Vähärautio A. (2025). Conserved cell state dynamics reveal targetable resistance patterns in ovarian high-grade serous carcinoma. Cancer Biology.

[78] Vázquez-García I., Uhlitz F., Ceglia N., Lim J.L.P., Wu M., Mohibullah N., Niyazov J., Ruiz A.E.B., Boehm K.M., Bojilova V. (2022). Ovarian cancer mutational processes drive site-specific immune evasion. Nature.

[79] Benjamin D., Sato T., Cibulskis K., Getz G., Stewart C., Lichtenstein L. (2019). Calling Somatic SNVs and Indels with Mutect2. Bioinformatics.

[80] Auwera G.A.V. de, O’Connor B.D. (2020).

[81] Karczewski K.J., Francioli L.C., Tiao G., Cummings B.B., Alföldi J., Wang Q., Collins R.L., Laricchia K.M., Ganna A., Birnbaum D.P. (2020). The mutational constraint spectrum quantified from variation in 141,456 humans. Nature.

[82] Sherry S.T., Ward M.H., Kholodov M., Baker J., Phan L., Smigielski E.M., Sirotkin K. (2001). dbSNP: the NCBI database of genetic variation. Nucleic Acids Res..

[83] Pruitt K.D., Brown G.R., Hiatt S.M., Thibaud-Nissen F., Astashyn A., Ermolaeva O., Farrell C.M., Hart J., Landrum M.J., McGarvey K.M. (2014). RefSeq: an update on mammalian reference sequences. Nucleic Acids Res..

[84] Landrum M.J., Chitipiralla S., Brown G.R., Chen C., Gu B., Hart J., Hoffman D., Jang W., Kaur K., Liu C. (2020). ClinVar: improvements to accessing data. Nucleic Acids Res..

[85] Tate J.G., Bamford S., Jubb H.C., Sondka Z., Beare D.M., Bindal N., Boutselakis H., Cole C.G., Creatore C., Dawson E. (2019). COSMIC: the Catalogue Of Somatic Mutations In Cancer. Nucleic Acids Res..

[86] Di Tommaso P., Chatzou M., Floden E.W., Barja P.P., Palumbo E., Notredame C. (2017). Nextflow enables reproducible computational workflows. Nat. Biotechnol..

[87] Amemiya H.M., Kundaje A., Boyle A.P. (2019). The ENCODE Blacklist: Identification of Problematic Regions of the Genome. Sci. Rep..

[88] Priestley P., Baber J., Lolkema M.P., Steeghs N., de Bruijn E., Shale C., Duyvesteyn K., Haidari S., van Hoeck A., Onstenk W. (2019). Pan-cancer whole-genome analyses of metastatic solid tumours. Nature.

[89] Shale C., Cameron D.L., Baber J., Wong M., Cowley M.J., Papenfuss A.T., Cuppen E., Priestley P. (2022). Unscrambling cancer genomes via integrated analysis of structural variation and copy number. Cell Genom..

[90] Koskela H., Li Y., Joutsiniemi T., Muranen T., Isoviita V.-M., Huhtinen K., Micoli G., Lavikka K., Marchi G., Hietanen S. (2024). HRD related signature 3 predicts clinical outcome in advanced tubo-ovarian high-grade serous carcinoma. Gynecol. Oncol..

[91] Sondka Z., Dhir N.B., Carvalho-Silva D., Jupe S., Madhumita null, McLaren K., Starkey M., Ward S., Wilding J., Ahmed M. (2024). COSMIC: a curated database of somatic variants and clinical data for cancer. Nucleic Acids Res..

[92] Ng P.C., Henikoff S. (2001). Predicting deleterious amino acid substitutions. Genome Res..

[93] Pankla R., Buddhisa S., Berry M., Blankenship D.M., Bancroft G.J., Banchereau J., Lertmemongkolchai G., Chaussabel D. (2009). Genomic transcriptional profiling identifies a candidate blood biomarker signature for the diagnosis of septicemic melioidosis. Genome Biol..

[94] Lin J.-R., Izar B., Wang S., Yapp C., Mei S., Shah P.M., Santagata S., Sorger P.K. (2018). Highly multiplexed immunofluorescence imaging of human tissues and tumors using t-CyCIF and conventional optical microscopes. eLife.

