# Supplementary material for: Multi-omics analysis reveals the attenuation of the interferon pathway as a driver of chemo-refractory ovarian cancer
Source: Cell Rep Med. 2025 Aug 29;6(9):102316. doi: 10.1016/j.xcrm.2025.102316 (PMC12490218; doi:10.1016/j.xcrm.2025.102316)
Supplement: Document S1. Figures S1–S8 and Tables S1, S2, S4, and S5 [file mmc1.pdf]

Cell Reports Medicine, Volume *6*

Supplemental information

Multi-omics analysis reveals the attenuation of the interferon pathway as a driver of chemo-refractory ovarian cancer

Daria Afenteva, Rong Yu, Anna Rajavuori, Marina Salvadores, Inga-Maria Launonen, Kari Lavikka, Kaiyang Zhang, Anna Pirttikoski, Giovanni Marchi, Sanaz Jamalzadeh, Veli-Matti Isoviita, Yilin Li, Giulia Micoli, Erdogan Pekcan Erkan, Matias M. Falco, Daniela Ungureanu, Alexandra Lahtinen, Jaana Oikkonen, Sakari Hietanen, Anna Vähärautio, Inderpreet Sur, Anni Virtanen, Anniina Färkkilä, Johanna Hynninen, Taru A. Muranen, Jussi Taipale, and Sampsa Hautaniemi

Figure S1. Flowchart of patient stratification from the DECIDER Clinical Trial. Related to Figure 1.

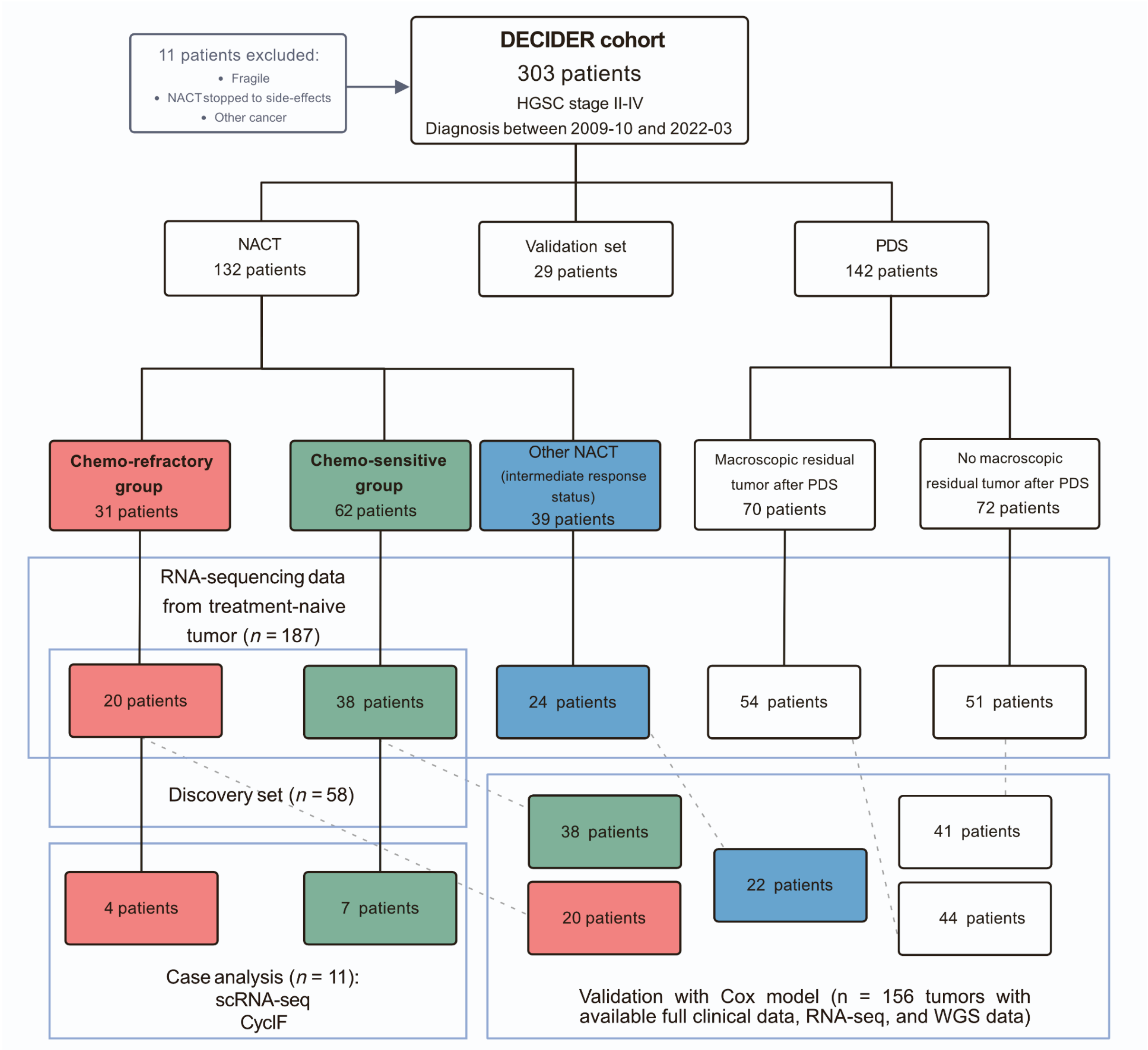

The chart illustrates the categorization of 303 high-grade serous carcinoma (HGSC) patients into 142 patients treated with primary debulking surgery (PDS) and 161 patients treated with neoadjuvant chemotherapy (NACT), excluding 11 patients due to various reasons, such as inadequate treatment or other cancers (Methods). The NACT group included patients with chemo-refractory (*n* = 31), intermediate (*n* = 39), and chemo-sensitive (*n* = 62) HGSC based on the response to NACT and platinum-free interval (PFI). Additionally, 29 NACT patients were included in the NACT validation set. The discovery set comprised bulk RNA-sequencing data from 20 patients with refractory cancer and 38 patients with sensitive cancer, supplemented by single-cell RNA-seq (scRNA-seq) and cyclic immunofluorescence (CycIF) for a subset of 11 patients. The set used for fitting the Cox proportional hazards model comprised NACT-treated patients with intermediate outcomes and patients who underwent PDS (*n* = 156).

Figure S2. Bulk RNA-seq data analysis reveals the downregulation of IFN-I activity in the chemo-refractory HGSC tumors. Related to Figure 3.

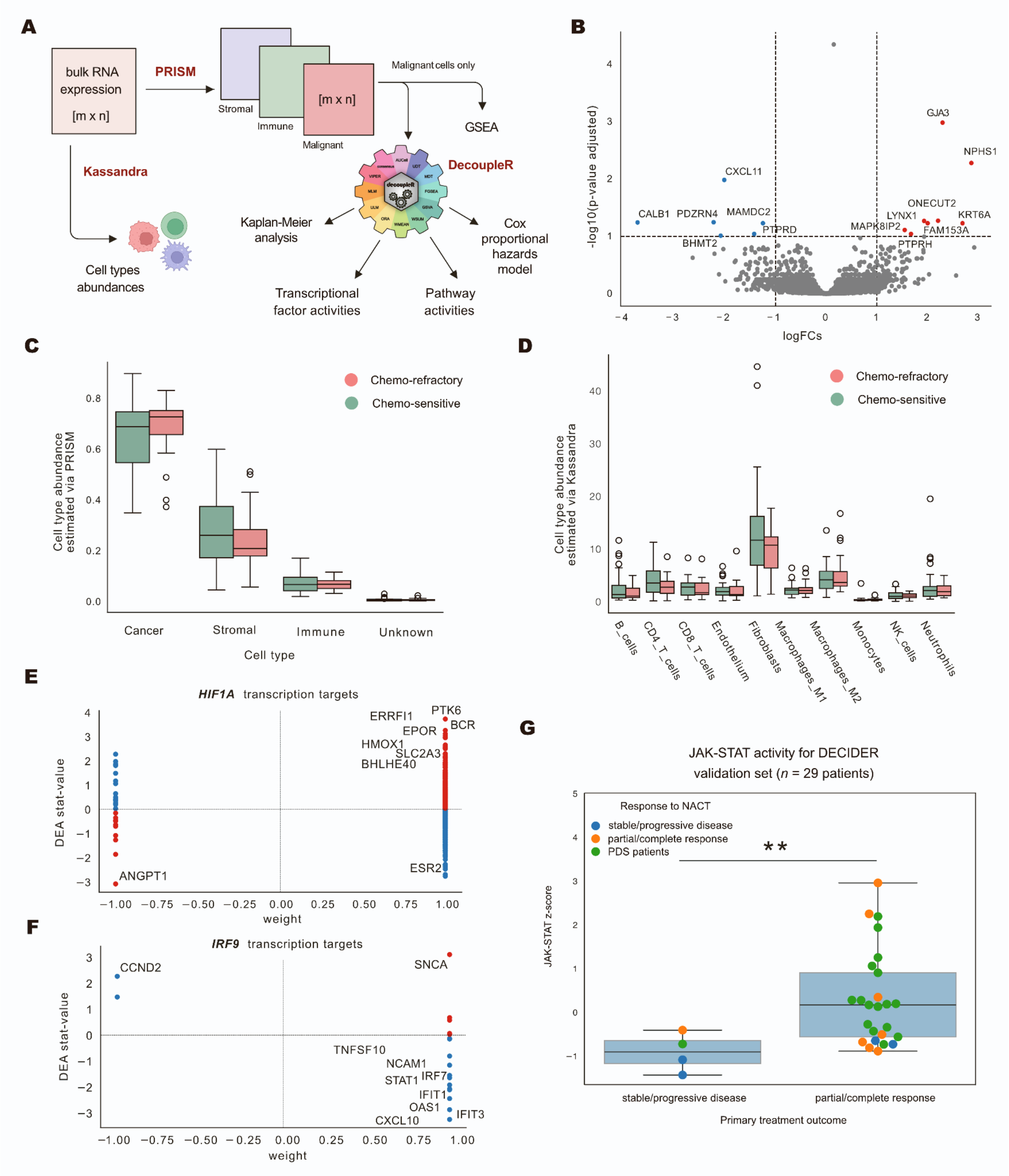

(A) Workflow diagram of the bulk RNA expression analysis of samples from chemo-refractory (n = 20) and chemo-sensitive (n = 38) tumors.

(B) The volcano plot of Differential Expression Analysis (DEA) between chemo-refractory (n = 20) and chemo-sensitive (n = 38) tumors results highlighting genes up-regulated (red) and down-regulated (blue) in the refractory tumors.

(C-D) Boxplot with comparison of cell type abundances between chemo-refractory (n = 20) and chemo-sensitive (n = 38) samples according to PRISM (C; Data S2) and Kassandra (D; Data S3). *P* values were

calculated by two-sided Student's t-test and adjusted by Benjamini-Hochberg (BH) FDR (no $P < 0.05$). Data are presented as median ± IQR (box: Q_1-Q_3; whiskers: furthest points within 1.5 x IQR).

(E, F) Scatter plots depicting the weight of the *HIF1A* (E) and *IRF9* (F) transcription target genes (x-axis) and their stat-value derived from the DEA (y-axis) of chemo-refractory versus chemo-sensitive tumors.

(G) JAK-STAT z-scores of pathway activity calculated from bulk RNA-seq of treatment-naive samples from the DECIDER validation set (n = 29 patients). Patients who exhibited stable or progressive disease (n = 4) after primary treatment were characterized by significantly lower JAK-STAT activity than those with partial or complete response (n = 25; two-tailed Student's t-test $P = 0.003$). Data are presented as median ± IQR (box: Q_1-Q_3; whiskers: furthest points within 1.5 x IQR). *$P < 0.05$, **$P < 0.01$, ***$P < 0.001$.

Figure S3. Genomic aberration analysis across the IFN-I signaling genes in chemo-refractory and chemo-sensitive HGSC. Related to Figures 2 and 3.

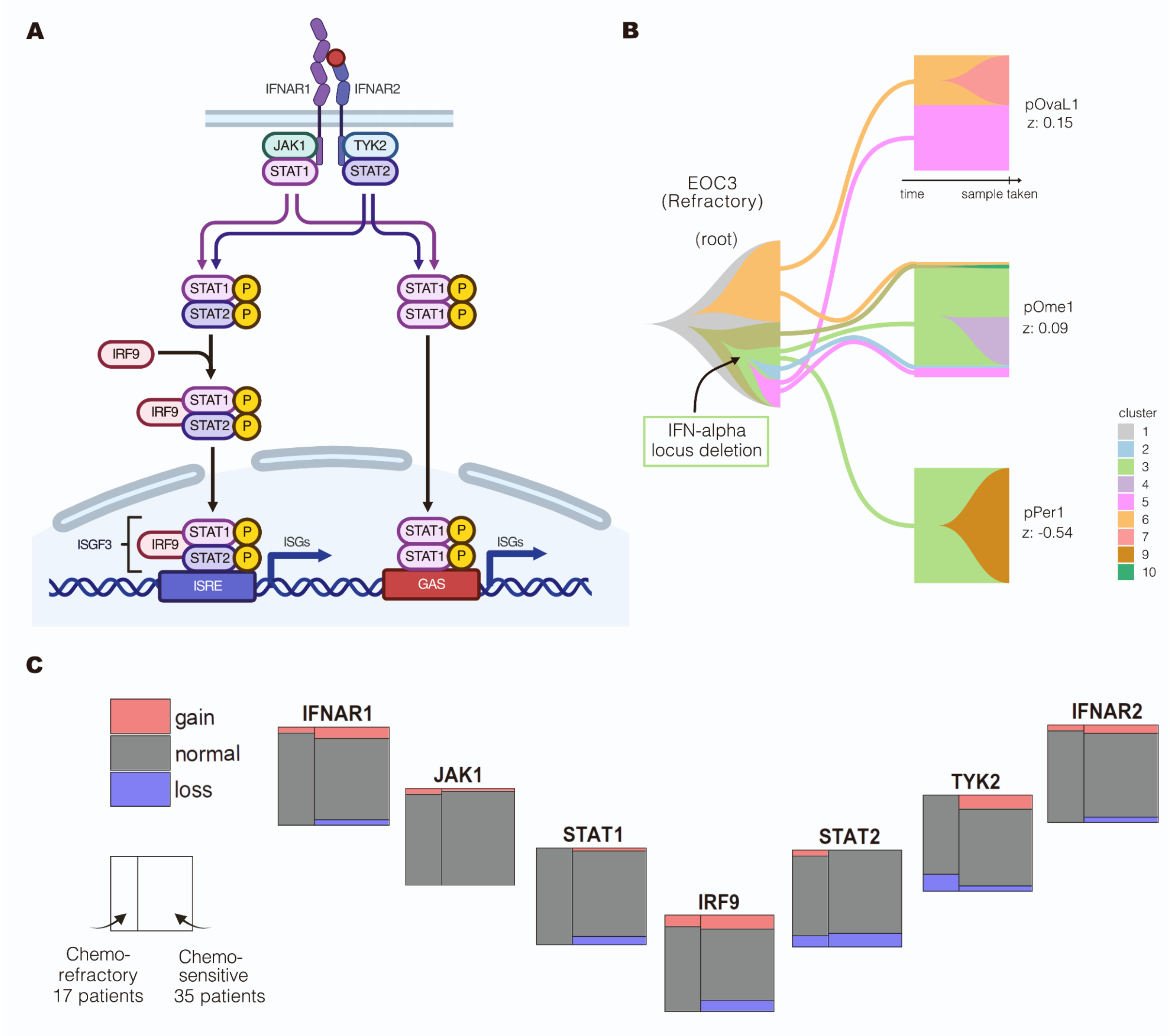

(A) Schematic representation of the IFN-I signaling pathway, illustrating the cascade from IFNAR1/2 activation through JAK/STAT phosphorylation and ending with IRF9-mediated interferon-stimulated genes (ISGs) transcription. ISGF3, interferon-stimulated gene factor-3; ISRE, interferon-stimulated response element; GAS, gamma-activated sequence.

(B) Jellyfish plot showing the clonal evolution across different samples in the chemo-refractory patient EOC3 harboring an IFN-alpha locus deletion, with colors indicating different subclones. The JAK-STAT z-scores for each sample are displayed as numbers.

(C) Mosaic plots showing genomic aberration analysis across the IFN-I signaling genes in chemo-refractory (n = 17) and chemo-sensitive (n = 35) HGSC tumors with matched bulk RNA-seq and WGS data.

Figure S4. Single-cell RNA-seq analyses confirm the downregulation of IFN-I response in cancer cells of chemo-refractory HGSC tumors. Related to Figure 4.

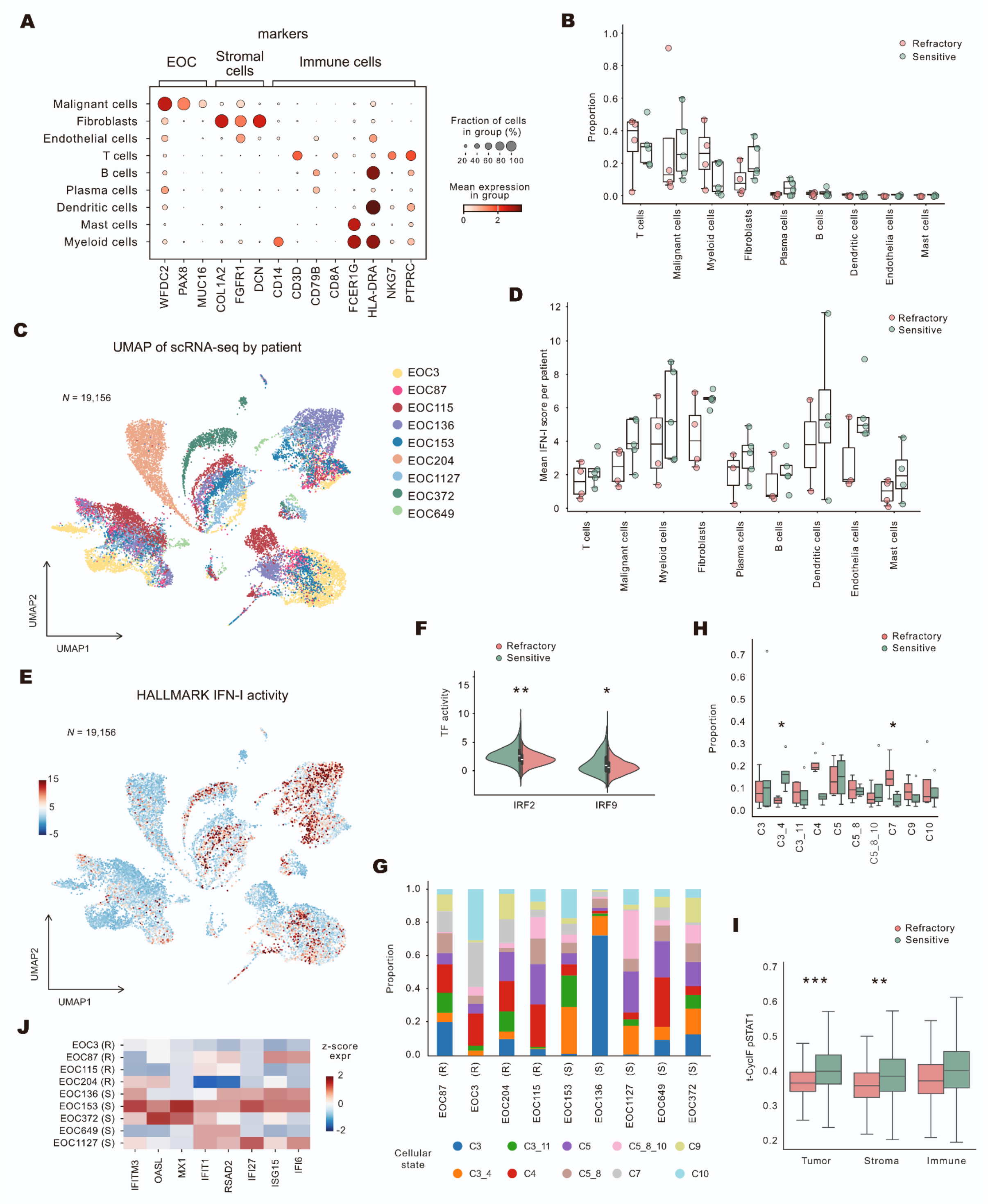

(A) Marker gene expression for different cell types, including 5,449 malignant, 3,232 stromal, and 10,475 immune cells across nine tumors. Expression levels are displayed as mean expression per group, with cell type-specific markers used to validate the annotation.

(B) Composition of scRNA-seq in chemo-sensitive (n = 5) and chemo-refractory (n = 4) tumors. P values were calculated by a two-sided Student's t-test and adjusted by Benjamini-Hochberg (BH) FDR (no $P < 0.05$;

adjusted values not shown). Data are presented as median ± IQR (box: Q_1-Q_3; whiskers: furthest points within 1.5 x IQR).

(C, E) UMAP plot of cells profiled by scRNA-seq colored by patient (C) and by IFN-I activity score (E).

(D) Comparison of mean IFN-I enrichment scores per patient across different cell types between four chemo-refractory and five chemo-sensitive tumors. *P* values were calculated by a two-sided Student's t-test and adjusted by BH FDR (no $P < 0.05$; adjusted values not shown). Data are presented as median ± IQR (box: Q_1-Q_3; whiskers: furthest points within 1.5 x IQR).

(F) Activity scores for transcription factors associated with IFN-I signaling (*IRF2* and *IRF9*) across chemo-refractory ($n = 4$ patients) and chemo-sensitive ($n = 5$ patients) tumors. *P* values were calculated by Generalized Estimating Equations (GEE) regression and adjusted by BH FDR.

(G) Proportions of cellular states defined by Zhang et al. within each tumor sample.

(H) Comparison of proportions of pre-defined cellular states between four chemo-refractory and five chemo-sensitive tumors. *P* values were calculated by a two-sided Student's t-test and adjusted by BH FDR. Data are presented as median ± IQR (box: Q_1-Q_3; whiskers: furthest points within 1.5 x IQR).

(I) t-CycIF analysis of pSTAT1 expression in single cells across tumor, stromal, and immune compartments between two chemo-refractory and two chemo-sensitive tumors. *P* values were calculated by GEE regression and adjusted by BH FDR. Data are presented as median ± IQR (box: Q_1-Q_3; whiskers: minimum and maximum). $^*P < 0.05$, $^{**}P < 0.01$, $^{***}P < 0.001$.

(J) Heatmap of z-scored-normalized expression for selected interferon-stimulated genes across nine HGSC tumors profiled with scRNA-seq (R = chemo-refractory, S = chemo-sensitive).

Figure S5. UMAP visualization of single-cell RNA-seq data used for cell type annotation. Related to Figure 4.

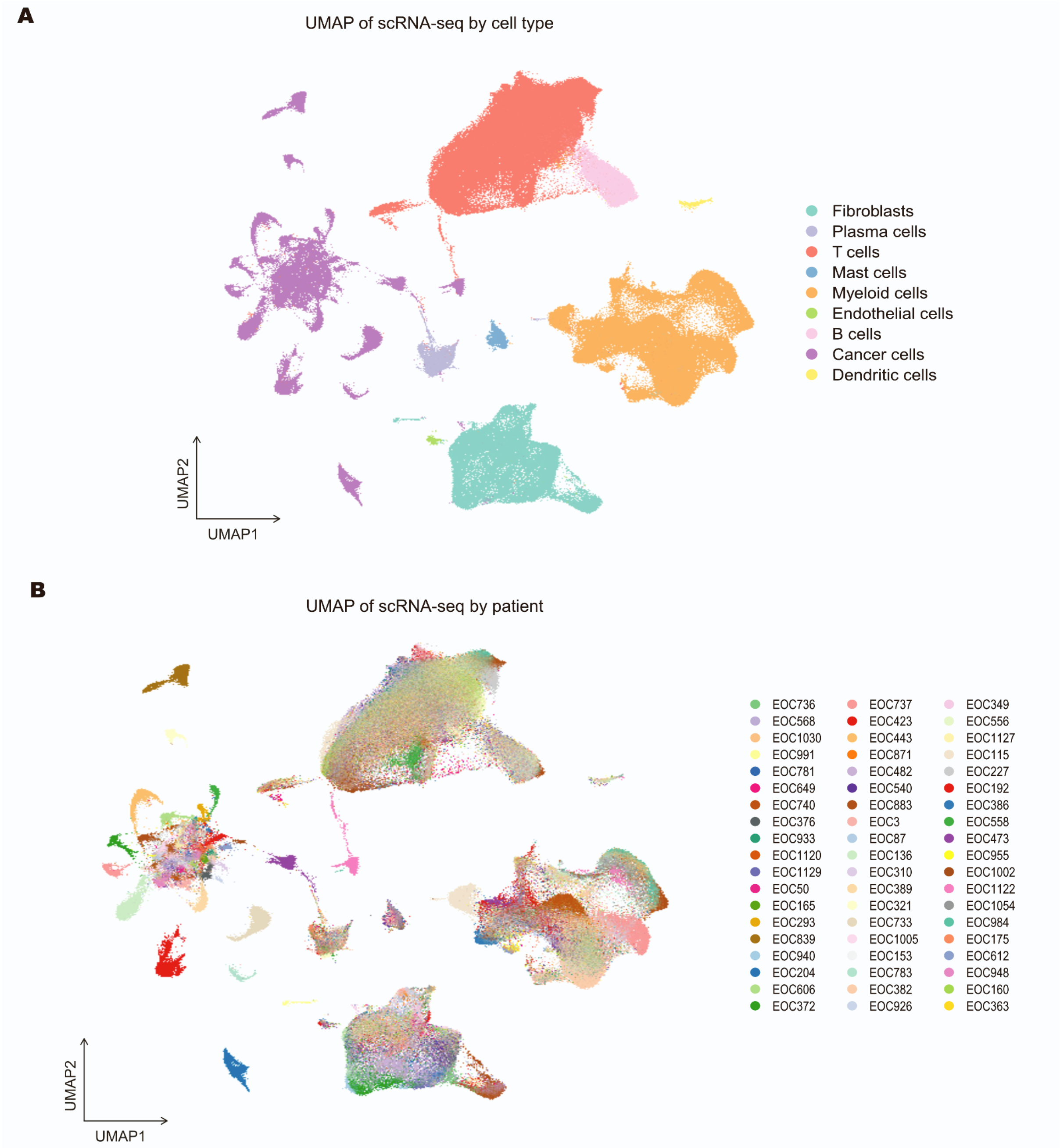

(A, B) UMAP plot of cells from 95 HGSC samples profiled by scRNA-seq, colored by cell type (A) and by patient (B).

Figure S6. Transcriptional and functional analyses in HGSC cell lines supporting the role of IFN-I response in platinum resistance. Related to Figure 5.

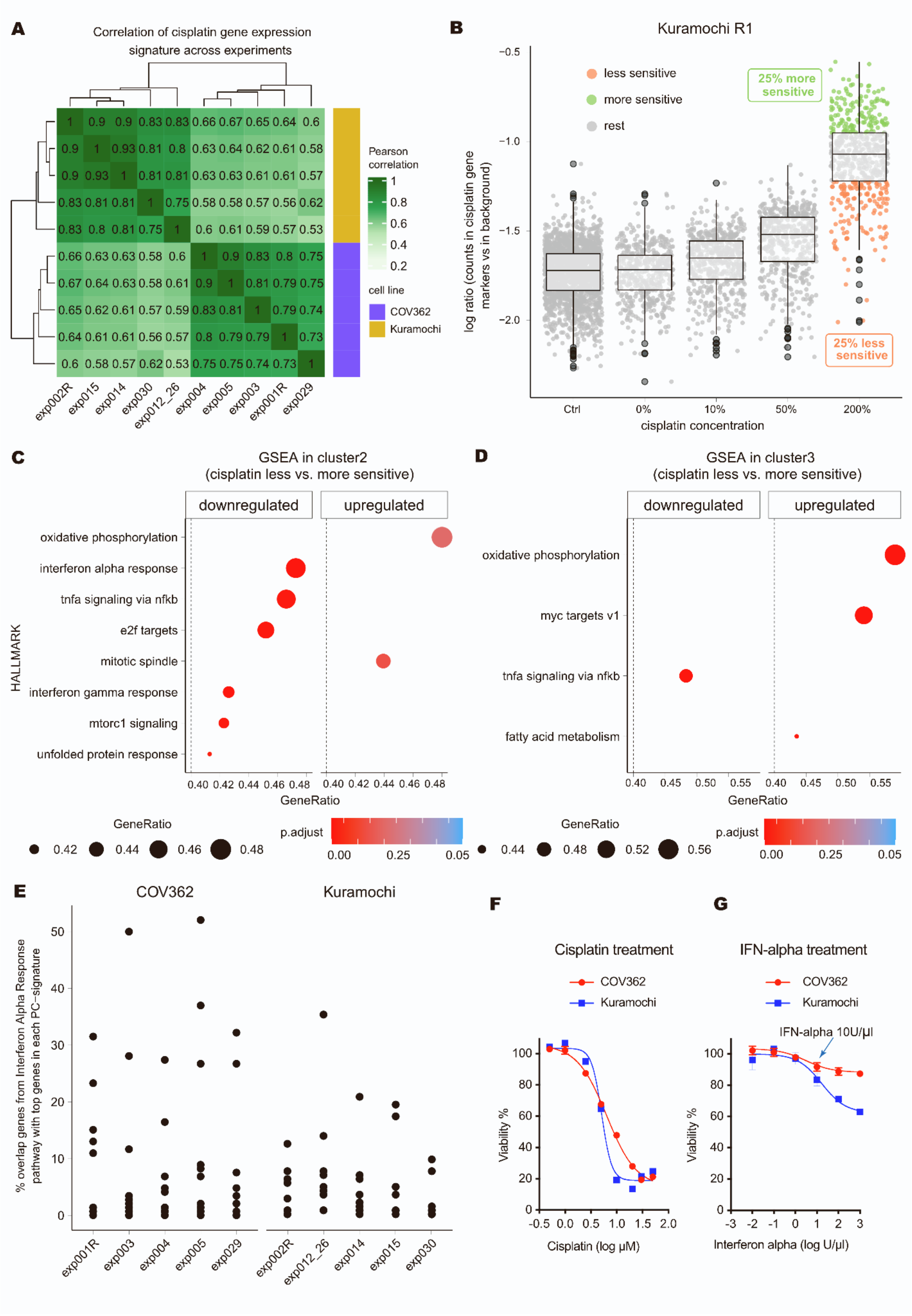

(A) Hierarchical clustering of the correlation between the transcriptional signatures (gene weights) obtained from 10 experiments of both COV362 and Kuramochi.

(B) Based on the CisSenScore, the treated cells were separated into ‘more-sensitive’ (top 25%, green) and ‘less-sensitive’ (bottom 25%, orange) in one experiment of Kuramochi cells treated with cisplatin. Data are presented as median ± IQR (box: Q_1-Q_3; whiskers: furthest points within 1.5 x IQR).

(C, D) GSEA scores for the Hallmark gene sets (with p.adjust < 0.05 and GeneRatio > 0.4) calculated based on the gene expression signatures in clusters 2 (C) and 3 (D) of the ‘less-sensitive’ cells compared with the ‘more-sensitive’ cells.

(E) Percentage of the 100 top genes in the Interferon Alpha Response pathway overlapping with top genes in each PC signature from the different experiments. Interferon Alpha Response variability signature was not recovered in Kuramochi cells due to their lack of baseline heterogeneity in the IFN-I response.

(F, G) Cell viability assay of cisplatin (F) and IFN-alpha (G) in ovarian cancer cells COV362 and Kuramochi.

Figure S7. Hierarchical clustering identifies the same 3 clusters across different thresholds. Related to Figure 5.

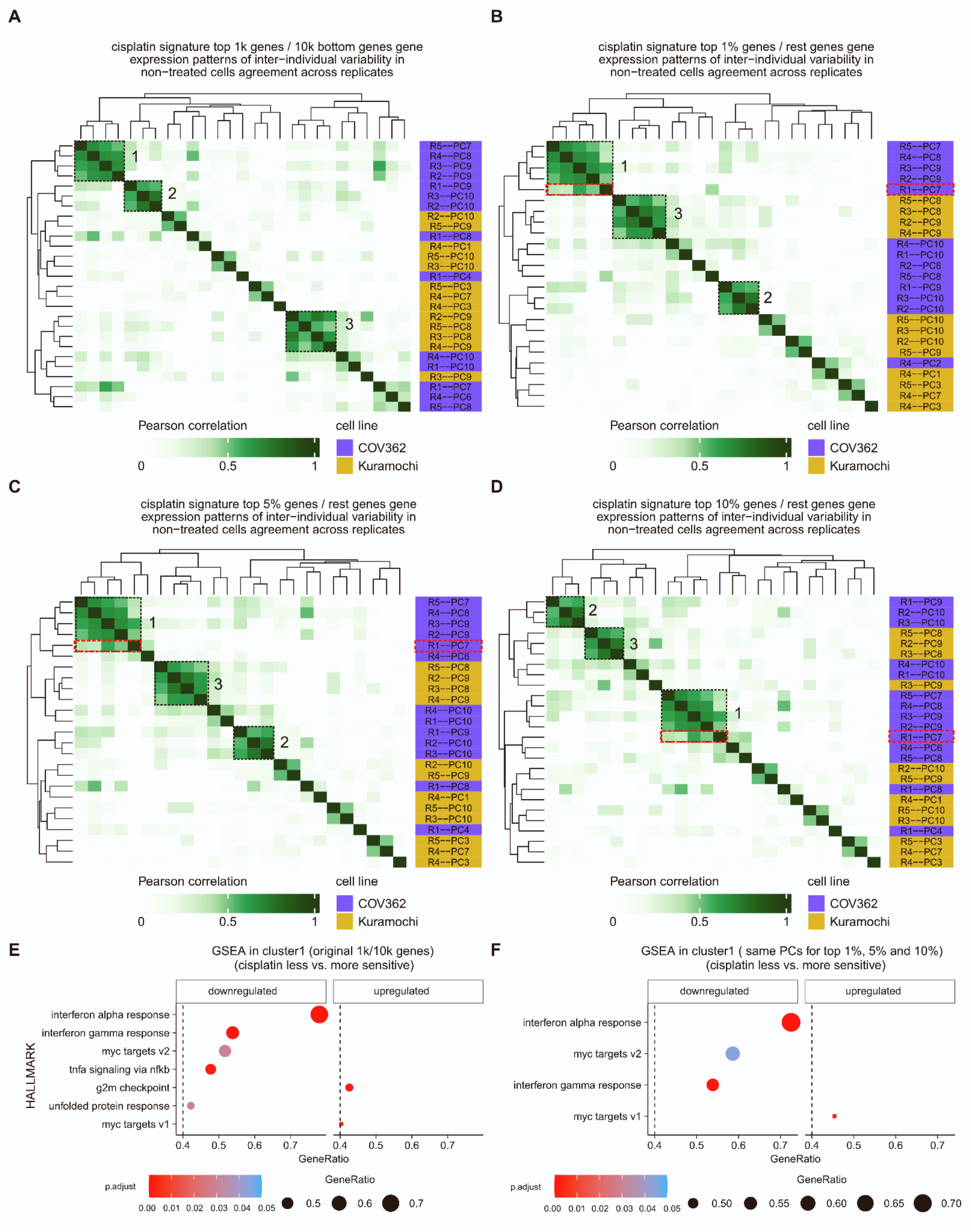

(A-D) Heatmap depicting pairwise similarities between gene expression patterns of inter-individual variability identified in non-treated cells across replicates using different thresholds: (A) Top 1k genes/ bottom 10k genes (identical to Figure 5E and used here for comparison purpose), (B) Top 1% genes / rest genes, (C) Top 5% genes / rest genes, (D) Top 10% genes / rest genes. The three common clusters across different thresholds are indicated

by black dashed boxes and numbers. R1–PC7, which is identified only in cluster 1 of 1%, 5% and 10% thresholds, is marked by red dashed boxes.

(E-F) GSEA scores for hallmark gene sets (with p.adjust < 0.05 and GeneRatio > 0.4) were calculated based on the gene expression signature in cluster 1 of the 'less-sensitive' cells compared with the 'more-sensitive' cells. Results from cluster 1 identify Interferon alpha response as the major determinant of cisplatin resistance, regardless of the threshold.

(E) GSEA results obtained using top 1% / bottom 10% genes (identical to Figure 5f and used here for comparison purposes)

(F) GSEA result representative of 1%, 5%, 10% thresholds.

Figure S8. Single-cell level expression of the top 10 genes in PC cluster 1 (from Figure 5E) in more- or less-sensitive cells to cisplatin treatment. Related to Figure 5.

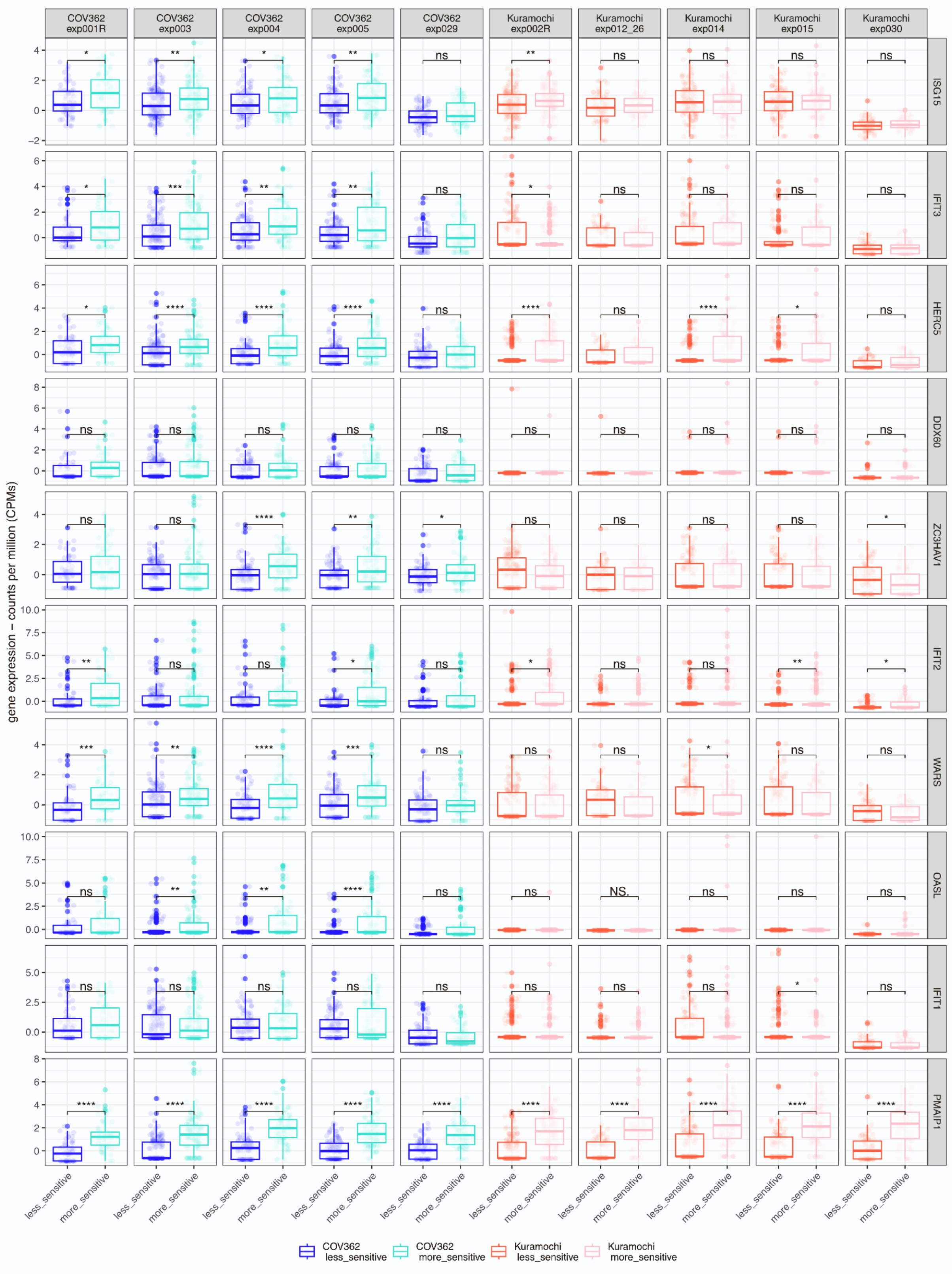

Five experimental replicates from COV362 and Kuramochi are shown. Data are presented as median ± IQR (box: Q_1-Q_3; whiskers: furthest points within 1.5 x IQR). *P* values were calculated by a Wilcoxon rank-sum test

(unadjusted *P* values are shown). *ns* represents no significant difference; **P* < 0.05; ***P* < 0.01; ****P* < 0.001; *****P* < 0.0001. Blue: COV362 cells less sensitive to cisplatin, Green: COV362 cells more sensitive to cisplatin, Orange: Kuramochi cells less sensitive to cisplatin, Pink: Kuramochi cells more sensitive to cisplatin.

Supplemental Tables

Table S1. Patient and tumor characteristics.

		**patients with chemo-refractory HGSC (*n* = 31)**	**patients with chemo-sensitive HGSC (*n* = 62)**	**p-value * (refractory vs. sensitive)**	**Patients with intermediate NACT-treated HGSC (*n* = 39)**	**PDS-treated patients (*n* = 142)**
**Diagnosis age**	median [range]	70 [51-85]	68 [38-86]	0.97	68 [54-83]	68.5 [39-88]
**Stage (FIGO2014)**	IIA-IIIA	0 (0%)	0 (0%)	0.9	0 (0%)	20 (14%)
	IIIB	0 (0%)	0 (0%)		1 (3%)	8 (6%)
	IIIC	20 (65%)	38 (61%)		27 (69%)	79 (55%)
	IVA	5 (16%)	13 (21%)		7 (18%)	8 (6%)
	IVB	6 (19%)	11 (18%)		4 (10%)	27 (19%)
**Dissemination score****	1-5	0 (0%)	3 (5%)	0.026	1 (3%)	37 (26%)
	6-10	7 (23%)	12 (19%)		8 (20.5%)	61 (43%)
	11-15	15 (48%)	34 (55%)		18 (46%)	38 (27%)
	>15	4 (13%)	0 (0%)		4 (10%)	4 (3%)
	unknown	5 (16%)	13 (21%)		8 (20.5%)	2 (1%)
**Over 1000 ml ascites at diagnosis**	No	6 (19%)	27 (44%)	0.024	14 (36%)	89 (63%)
	Yes	25 (81%)	35 (56%)		25 (64%)	50 (35%)
	unknown	0 (0%)	0 (0%)		0 (0%)	3 (2%)
**SBS3 mutational signature**	HRD	11 (35%)	28 (45%)	0.35	10 (26%)	56 (39%)
	HRP	17 (55%)	26 (42%)		20 (51%)	45 (32%)
	unknown	3 (10%)	8 (13%)		9 (23%)	41 (29%)
**ID6 mutation signature**	HRD	7 (23%)	25 (40%)	0.20	4 (10%)	55 (39%)
	HRP	16 (52%)	26 (42%)		24 (62%)	62 (44%)
	unknown	8 (26%)	11 (18%)		11 (28%)	25 (17%)
**HR gene mutation**	No	20 (65%)	38 (61%)	0.65	26 (67%)	86 (61%)
	Yes	3 (9%)	14 (23%)		2 (5%)	32 (82%)
	unknown	8 (26%)	10 (16%)		11 (28%)	24 (17%)
**Foldback inversions**	Not detected	13 (42%)	23 (37%)	0.45	14 (36%)	60 (42%)
	Detected	10 (32%)	28 (45%)		14 (36%)	58 (41%)
	unknown	8 (26%)	11 (18%)		11 (28%)	24 (17%)
**Any debulking surgery**	Yes	11 (35%)	60 (97%)	1.3e-10	33 (85%)	142 (100%)
	No	20 (65%)	2 (3%)		6 (15%)	0 (0%)
**Residual**	No visible	4 (13%)	18 (29%)	1.7e-06	15 (38%)	72 (51%)

**tumor after debulking surgery**	tumor					
	1 to 10mm	6 (19%)	35 (56%)		14 (36%)	46 (32%)
	more than 10mm	21 (68%)	9 (15%)		10 (26%)	24 (17%)

* The refractory and reference groups were compared with the Mann-Whitney test for diagnosis age and the Fisher exact test for other characteristics.

** Isoviita et al. 2019

Table S2. Genomic characteristics of Kuramochi and COV362 cell lines.

Cell line	TP53	BRCA1	BRCA2	RB1	MYC
**Kuramochi**	Mutation		Mutation		Amplification
**COV362**	Mutation	Mutation		Low mRNA expression	Amplification

Table S4. Raw scRNA-seq samples availability in EGA.

Sample	Previous_publication_DOI	EGA_study_accession_number
EOC87_pPer	https://www.science.org/doi/epdf/10.1126/sciadv.abm1831	EGAS00001005010
EOC3_pPer	https://www.science.org/doi/epdf/10.1126/sciadv.abm1831	EGAS00001005010
EOC204_pMes	NA	EGAS00001005010
EOC115_pPer	NA	EGAS00001005010
EOC153_pOme	https://www.science.org/doi/epdf/10.1126/sciadv.abm1831	EGAS00001005010
EOC136_pMes	https://www.science.org/doi/epdf/10.1126/sciadv.abm1831	EGAS00001005010
EOC1127_pOme	NA	EGAS00001005010
EOC649_pOme	NA	EGAS00001005010
EOC372_pPer	https://www.science.org/doi/epdf/10.1126/sciadv.abm1831	EGAS00001005010

Table S5. Oligonucleotides used in the study (supplemental to the Key Resource Table).

REAGENT OR SOURCE	SOURCE	IDENTIFIER
/5AmMC6/GTGACTGGAGTTCAGACGTGTGCTCTTCCGATCTNNNNNNNNNNGTCAACTCTTTAGCGNNNNNNNNNGCTTTAAGGCCGGTCCTAGC*A*A	IDT	Sequence-based
/5AmMC6/GTGACTGGAGTTCAGACGTGTGCTCTTCCGATCTNNNNNNNNNNTGATGGCCTATTGGGNNNNNNNNNGCTTTAAGGCCGGTCCTAGC*A*A	IDT	Sequence-based
/5AmMC6/GTGACTGGAGTTCAGACGTGTGCTCTTCCGATCTNNNNNNNNNNTTCCGCCTCTCTTTGNNNNNNNNNGCTTTAAGGCCGGTCCTAGC*A*A	IDT	Sequence-based
/5AmMC6/GTGACTGGAGTTCAGACGTGTGCTCTTCCGATCTNNNNNNNNNNAGTAAGTTCAGCGTANNNNNNNNNGCTTTAAGGCCGGTCCTAGC*A*A	IDT	Sequence-based
/5AmMC6/GTGACTGGAGTTCAGACG	IDT	Sequence-based

TGTGCTCTTCCGATCTNNNNNNNNNNAAGTATCGTTTCGCANNNNNNNNNNGCTTTAAGGCCGGTCCTAGC*A*A		
/5AmMC6/GTGACTGGAGTTCAGACGTGTGCTCTTCCGATCTNNNNNNNNNNGGTTGCCAGATGTCANNNNNNNNNNGCTTTAAGGCCGGTCCTAGC*A*A	IDT	Sequence-based
/5AmMC6/GTGACTGGAGTTCAGACGTGTGCTCTTCCGATCTNNNNNNNNNNTGTCTTTCCTGCCAGNNNNNNNNNNGCTTTAAGGCCGGTCCTAGC*A*A	IDT	Sequence-based
/5AmMC6/GTGACTGGAGTTCAGACGTGTGCTCTTCCGATCTNNNNNNNNNNCTCCTCTGCAATTACNNNNNNNNNNGCTTTAAGGCCGGTCCTAGC*A*A	IDT	Sequence-based
/5AmMC6/GTGACTGGAGTTCAGACGTGTGCTCTTCCGATCTNNNNNNNNNNACCCACCAGTAAGACNNNNNNNNNNGCTTTAAGGCCGGTCCTAGC*A*A	IDT	Sequence-based
/5AmMC6/GTGACTGGAGTTCAGACGTGTGCTCTTCCGATCTNNNNNNNNNNGGTCGAGAGCATTCANNNNNNNNNNGCTTTAAGGCCGGTCCTAGC*A*A	IDT	Sequence-based
/5AmMC6/GTGACTGGAGTTCAGACGTGTGCTCTTCCGATCTNNNNNNNNNNCTTGCCGCATGTCATNNNNNNNNNNGCTTTAAGGCCGGTCCTAGC*A*A	IDT	Sequence-based
/5AmMC6/GTGACTGGAGTTCAGACGTGTGCTCTTCCGATCTNNNNNNNNNNAAAGCATTCTTCACGNNNNNNNNNNGCTTTAAGGCCGGTCCTAGC*A*A	IDT	Sequence-based
/5AmMC6/GTGACTGGAGTTCAGACGTGTGCTCTTCCGATCTNNNNNNNNNNCTTTGTCTTTGTGAGNNNNNNNNNNGCTTTAAGGCCGGTCCTAGC*A*A	IDT	Sequence-based
/5AmMC6/GTGACTGGAGTTCAGACGTGTGCTCTTCCGATCTNNNNNNNNNNTATGCTGCCACGGTANNNNNNNNNNGCTTTAAGGCCGGTCCTAGC*A*A	IDT	Sequence-based
